# Investigating the complex story of one ditch—A multidisciplinary study of ditch infill provides insight into the spatial organisation within the oppidum of Bibracte (Burgundy, France)

**DOI:** 10.1371/journal.pone.0231790

**Published:** 2020-04-20

**Authors:** Petra Goláňová, Mária Hajnalová, Lenka Lisá, Peter Milo, Libor Petr, Markéta Fránková, Jan Kysela, Patrik G. Flammer, Romana Kočárová, Peter Barta

**Affiliations:** 1 Department of Archaeology and Museology, Faculty of Arts, Masaryk University, Brno, Czech Republic; 2 Department of Archaeology, Faculty of Arts, Constantine the Philosopher University, Nitra, Slovak Republic; 3 Institute of Geology, Czech Academy of Sciences, Prague, Czech Republic; 4 Department of Archaeology and Museology, Faculty of Arts and Department of Botany and Zoology, Faculty of Science, Masaryk University, Brno, Czech Republic; 5 Department of Vegetation Ecology, Laboratory of Paleoecology, Czech Academy of Sciences, Brno, Czech Republic; 6 Institute of Classical Archaeology, Faculty of Arts, Charles University, Prague, Czech Republic; 7 Department of Zoology, University of Oxford, Oxford, England, United Kingdom; 8 Independent Researcher, Brno, Czech Republic; 9 Department of Archaeology, Faculty of Arts, Comenius University in Bratislava, Bratislava, Slovak Republic; University at Buffalo - The State University of New York, UNITED STATES

## Abstract

Seemingly empty spaces in various archaeological settings have left many unanswered questions. This paper focuses on the appearance, maintenance and possible function of a large empty area situated at the summit plateau of the Iron Age oppidum Bibracte in France. Multidisciplinary research of the infill of the ditch that delimited this area in the 1^st^ century BC has provided evidence on the primary function and the formation processes of the structure itself, and for the reconstruction of the appearance, maintenance and function of the area it enclosed. The results allow us to gain insight into a variety of topics, including the role of trees, hygiene measures and waste management strategies at this urbanised hilltop centre. This paper demonstrates that multi-proxy analyses provide detailed insight into the function of archaeological features in a local environmental context and the potential of such approaches in archaeology.

## Introduction

We present a multidisciplinary approach to investigate a ditch that crosses an open space within the oppidum of Bibracte, an important fortified Late Iron Age site in France.

The archaeological excavations of oppida—the fortified urbanized centres spanning the Late Iron Age to Early Roman transition—almost exclusively focus on the originally built-up areas. Projects studying the entire enclosed area as one urban space, and thus also looking at the role and function of seemingly ‘empty’ areas, are rare (cf. e.g., Manching: [1]; cf. a summary in [2]). The extensive use of geophysics at these sites during the last decade has substantially extended the knowledge gained through excavations [3–7]. The possible structures detected during these surveys have only rarely been verified by subsequent archaeological excavations, and no special attention has been paid to the areas where no traces of buildings have been found [5, 8].

‘Empty spaces’ within urban centres are deliberately not built up and are maintained as such for a prolonged period of time. The relatively small areas, which are surrounded by buildings and are sometimes paved, are generally identified as public spaces (‘squares’) intended for gathering or interaction, e.g., at Manching [9, 10] and Corent [11, 12]. Less clear is the function of large areas within the oppida with almost no traces of human activities. Several possible functions have been suggested for their role, such as use as fields, pastures, space for social events (gatherings, marketplaces), refuge or spare land for urban development [2, 13–15]. At some oppida, scattered farming tools were recovered mostly during metal detector surveys alongside a low density of reworked/eroded artefacts attributed to household waste found at the top-soil horizons during excavation. This evidence suggests that these large ‘empty/open spaces’ were used as grazing or arable land [7, 15, 16]. Furthermore, the integration of agricultural production inside the ramparts at the oppida is considered as one of the key features of low-density urbanism [17]. In the case of Manching, a lowland oppidum (terminology by V. Salač [18]), the rampart enclosed the entire area of a formerly open settlement, including fields and pastures. This interpretation is supported by the numerous seeds of arable weeds recovered from drainage channels delimiting the fields. Plant remains from the built-up areas differed substantially, consisting mainly of stored and milled crops [19]. However, conclusive evidence of agricultural use of the empty spaces is to date missing for hilltop oppida. This study presents a multidisciplinary approach for the examination of such a large empty space by studying the fill of the associated sunken structure(s)–in this case the delimiting ditch.

Ditches at Late Iron Age fortified sites, like hillforts and oppida, were usually part of complex fortification structures. Generally situated outside the ramparts, these structures mainly had the strategic function to improve defences. However, similar structures are sometimes also present *intra muros*, delimiting or separating spaces with special functions (e.g., Villeneuve-Saint-Germain, Titelberg). While most studies focus on the area they delimit and concentrate on establishing the chronology of ditch infills based on artefacts, the ditch sediments themselves are often neglected as a valuable source of information on the surrounding space and formation processes [13]. The rare scientific analyses that are available are mainly based on plant macro-remains and pollen, and in some cases also animal bones [19, 20].

The studied ditch in Bibracte enclosed a seemingly empty space, whose maintenance or function has never been studied by appropriate methods. During the excavation of the Bibracte ditch, we applied different methods to obtain complex information alongside the relatively common archaeobotany, anthracology and palynology analyses. Besides macroscopical observations, methods such as microstratigraphical description [21–23], geochemistry [24, 25] and proxies, including diatoms [26, 27] and parasitology [28] contribute valuable information. Radiocarbon dating also provided useful evidence, despite the relatively short period over which the ditch was filled in. No previous study has combined so many different analytical techniques on one structure in an Iron Age oppidum.

The oppidum of Bibracte, situated on Mont Beuvray at 821 m a.s.l. (near Autun, Burgundy, France), is a well-known site that was described in Caesar’s Gallic Wars as the capital of the Celtic tribe of the Aedui. It has been subject to intense archaeological research since the 19^th^ century, although excavations were discontinued in 1914. In 1984, excavations were resumed and have continued since then [29, 30]. This commercial, cultural and political centre was, according to Caesar’s description [31], ‘by far the largest and the best-provided of the Aeduan towns’. It was founded at the end of the 2^nd^ century BC. Extensive geophysical surveys have contributed significantly to our understanding of the structure and urbanism at Bibracte [30]. The documented changes in architecture and material culture reflect the political and cultural shifts that took place during its brief existence. Bibracte reached its peak in the second half of the 1^st^ century BC, shortly before its rapid abandonment in favour of a new city, Augustodunum (Autun). In the Middle Ages, settlement activities on Mont Beuvray were limited to the Franciscan monastery erected in the 14^th^ century AD and to the area on the summit plateau of La Chaume within the sacral/religious zone near a St. Martin chapel built in the area of a fanum from the 1^st^– 3^rd^ century AD. Annual fairs took place on the summit plateau at least since the Medieval period [32, 33].

**Fig 1:**
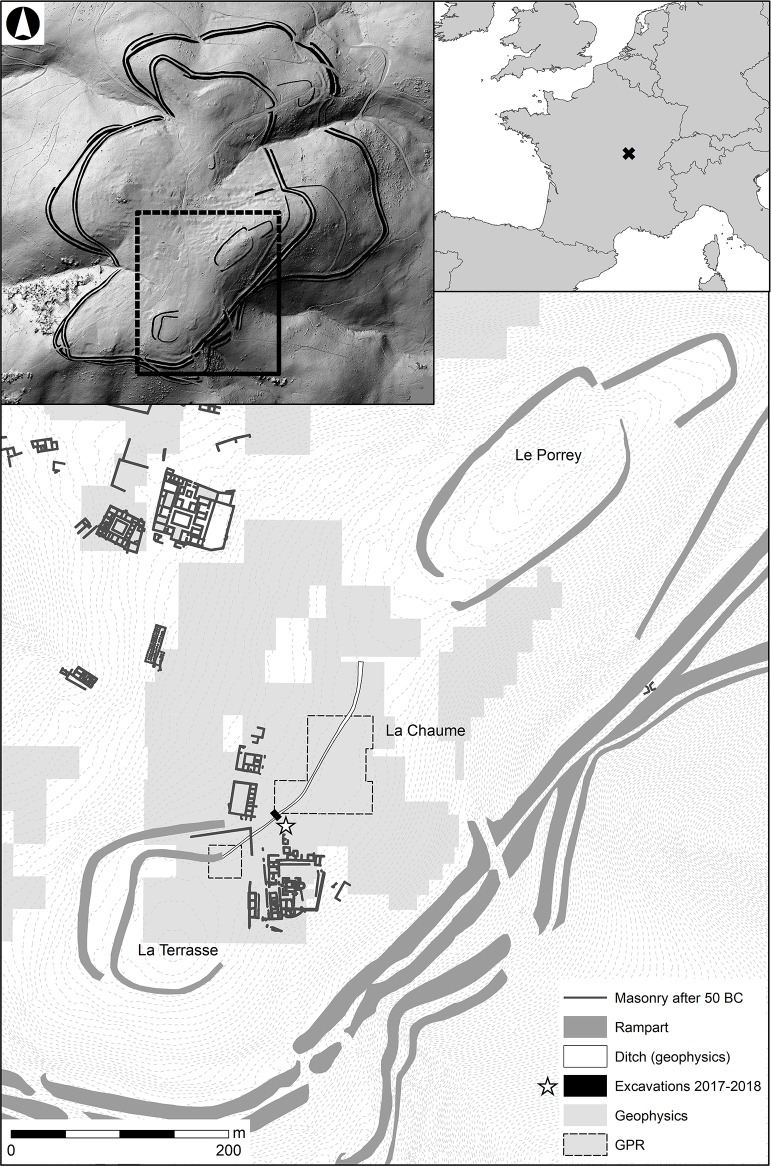
Topographic map of Bibracte oppidum/Mont Beuvray. Details of La Terrasse-Le Porrey area with the locations of the surveyed and excavated areas.

The oppidum was initially surrounded by a long rampart system (7 km) that was soon abandoned in favour of a shorter one (5.2 km). The shorter but stronger fortification, with preserved rampart heights of up to 4 metres, is contrasted by enclosure systems discovered *intra muros*. The strategic use of these internal structures is debated [34]. The inner earthworks, still visible at the summits of La Terrasse and Le Porrey (Fig 1), were supplemented by one deep and a number of shallow ditches [35, 36]. On the plateau of La Chaume, which connects both summits and offers the best view on the surrounding landscape, the ditch (linked to the fortification of La Terrasse) was discovered based on geophysical surveys, which were followed in 2017–2018 by targeted excavations of the ditch infill. Previous excavations on the summit plateau of La Chaume did not reveal any archaeological structures connected to or contemporaneous with the oppidum occupation phase.

The original aim of our project was to verify the geophysical interpretation of the ditch and to determine the construction date. However, the presence of waterlogged deposits in the lower part of the ditch fill raised a number of new questions. These waterlogged deposits were interpreted as a probable source of in situ sealed palaeoecological data, and thus, different sample assemblages were immediately collected to address research questions on the historical environment. These questions aimed foremost at a better understanding of the fill formation processes of the ditch and a reconstruction of the local environment at the summit plateau during the use of the ditch and shortly after. The targeted aims were as follows: 1. determine the presence/absence of water in the ditch during its function and if present, specify its quality; 2. carry out absolute chronological dating (AMS) of fill layers; 3. reconstruct the vegetation cover; 4. determine the possible land-use of the surrounding area (e.g., forest/woodland/grassland/arable land) and the degree of human and/or animal impact; 5. reconstruct the ditch backfill processes; 6. identify the character and use of the area shortly after the ditch infill processes were complete. This innovative approach involving a variety of methods to investigate the contents of a ditch or other sunken features could be used as a framework for further research and highlights the value of multidisciplinary research approaches in archaeology.

## Methods

### Geophysical survey

The aim of the geophysical survey at Bibracte was the localization and identification of all potential archaeological structures. The discovery of the ditch dates back to 2012, when the survey of La Chaume sector began. Magnetometry and georadar surveys were applied in this study.

The Fluxgate magnetometer Ferex (Förster) was used for the magnetic research. The instrument is designed as a gradiometer, has four probes, and is capable of recording magnetic field strength values with an accuracy of 0.1 nT/m. The measurements were carried out in a network of interconnected polygons of 30 × 30 m. The density of the measured points was 0.25 × 0.5 m. Foerster Dataload and Magdatashift software were used for data processing. The resulting magnetogram was created in Surfer (Golden software Inc.) software. RAMAC X3M (Geoscience Malå) with shielded antennas of 250 MHz and 500 MHz frequencies was used for the georadar survey. The measurement was performed along the same network as the magnetometry. The density of the measured points was 0.1 × 0.5 m. The measurements were recorded in radarograms as vertical and horizontal time slices. RAMAC Ground Vision software was used for data processing. The spatial representation of the surveyed areas was processed using Easy 3D and Archeo Fusion software.

### Archaeology

**Fig 2:**
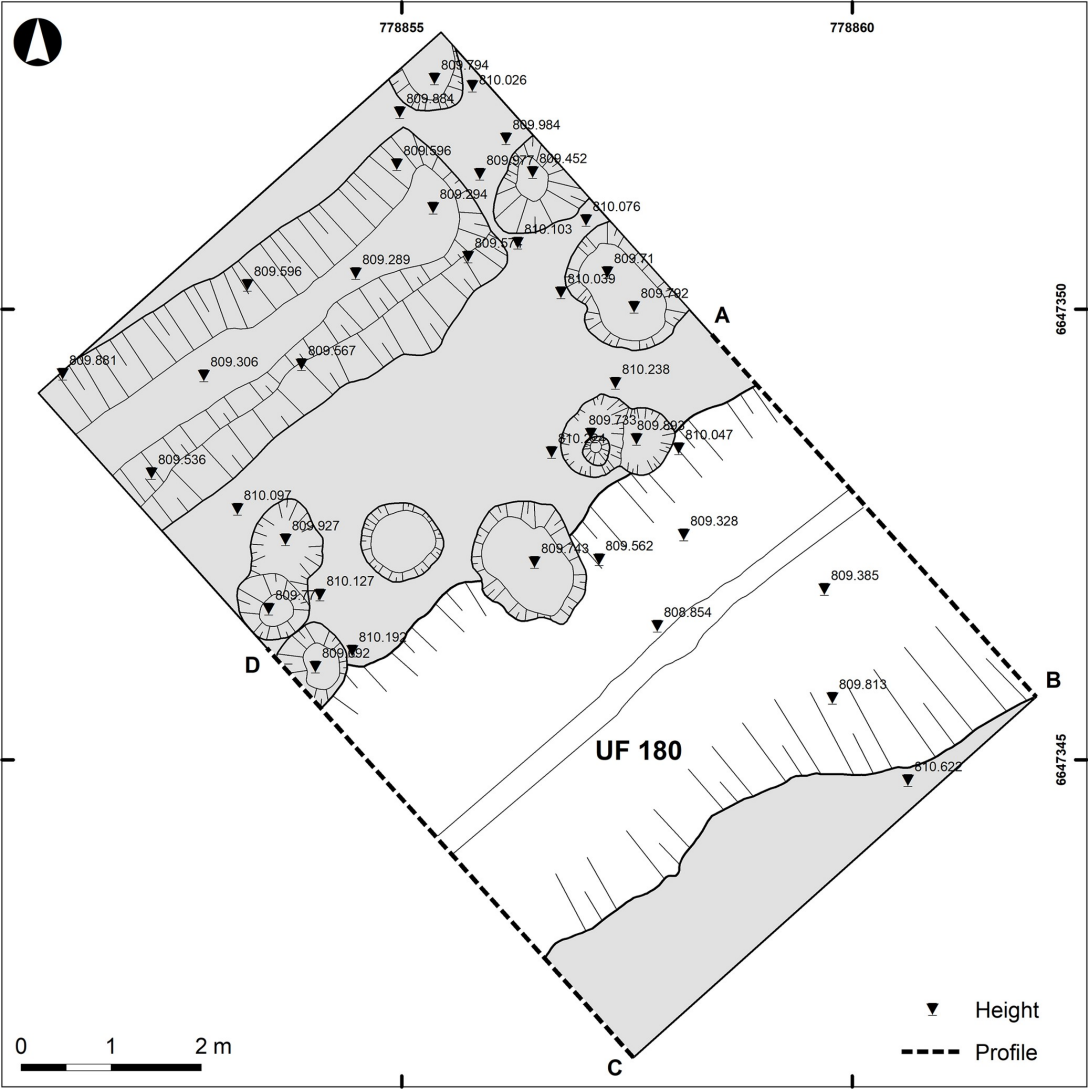
Bibracte/Mont Beuvray—La Chaume. Plan of the excavated part of ditch UF180 with the location of the ditch sections (see Figs 3 and 4).

**Fig 3:**
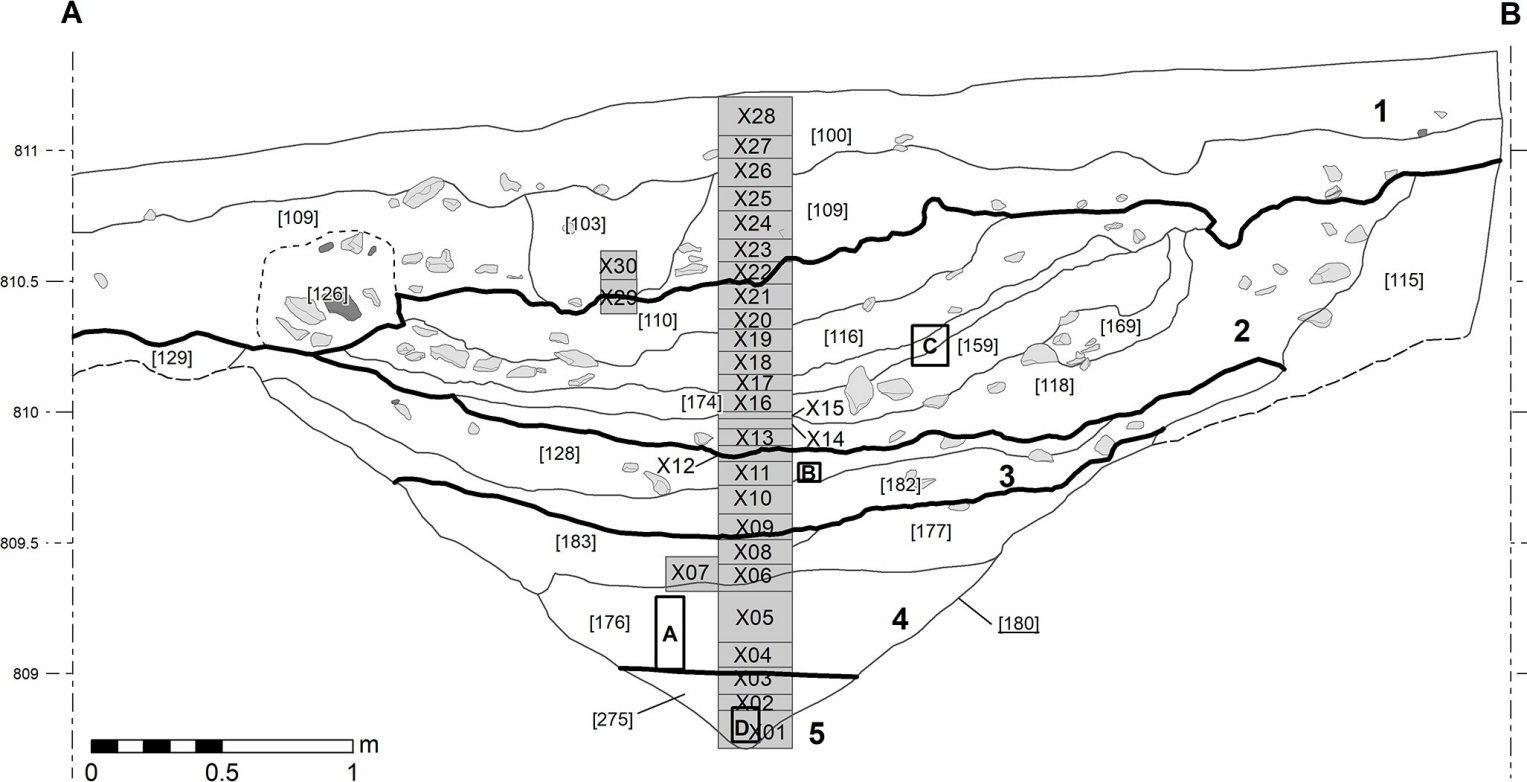
Bibracte/Mont Beuvray—La Chaume (ditch UF180). North-east section with the location of samples X1-X28 and micromorphological sampled positions A-D.

**Fig 4:**
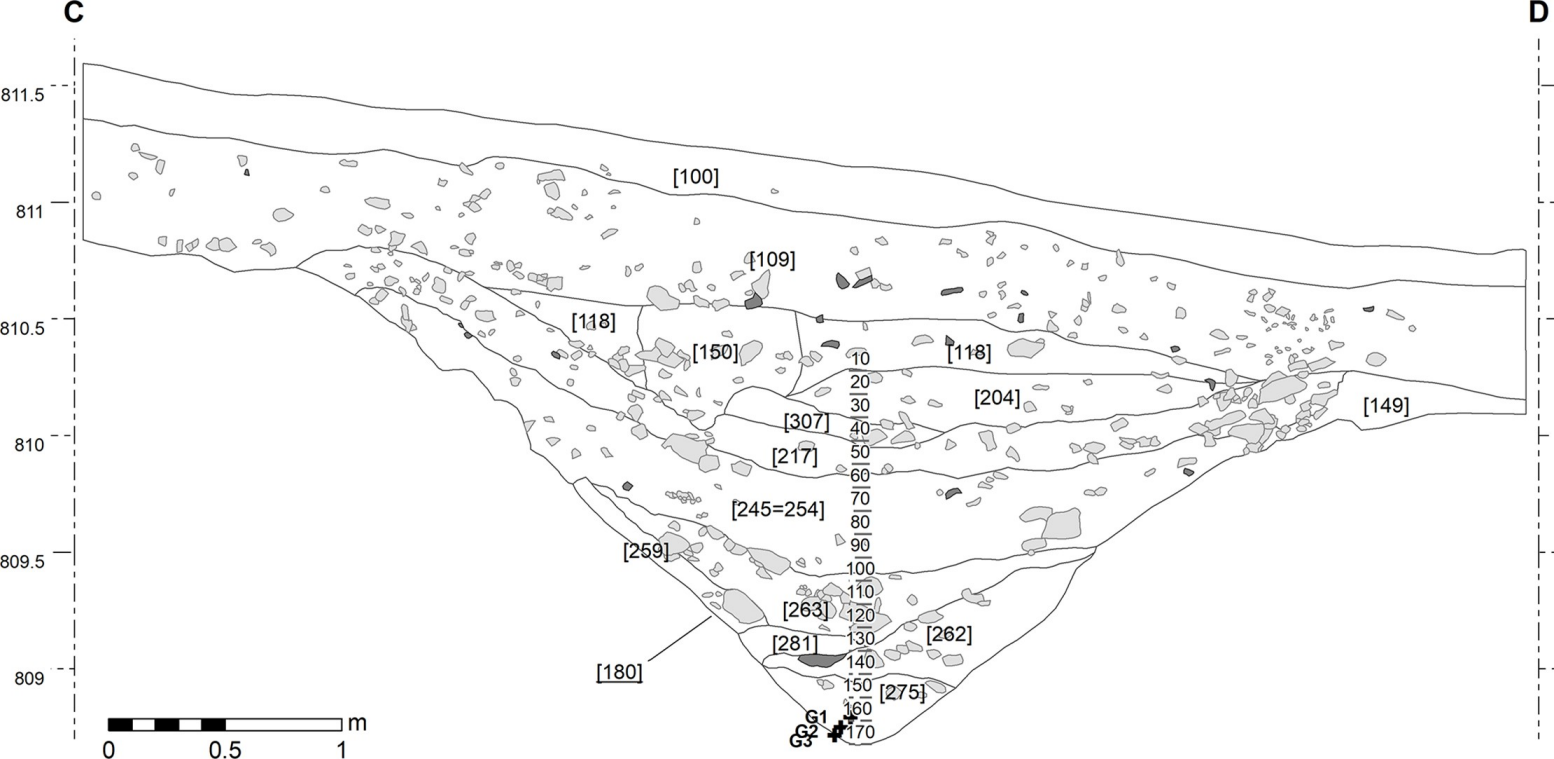
Bibracte/Mont Beuvray—La Chaume (ditch UF180). South-west section with the location of samples G1-3.

A trench measuring 6 × 10 m was opened to investigate the results of the geophysical survey (Fig 2). The excavation was systematically documented with Total Station and photogrammetry. All metal finds were precisely located. The Harris matrix was applied, showing the connections between individual contexts. Only the grass turf was removed using a mechanical excavator and open area excavation was performed for the upper levels. Once the limits of the ditch were detected at a depth of 65 cm, the excavation continued in one half of the trench deposits following the stratigraphic layers (Fig 3). The second half of the ditch fill was excavated in 2018 applying a more detailed approach (Fig 4). The deposits in the ditch were excavated in 10 cm layers within a grid of 50 × 50 cm, respecting their relationship to the actual stratigraphic units. The volume of these analytical units was measured (in litres of soil), and the volume and weight (in kilograms) of stones was assessed in seven of these squares using dry sieving (mesh size 5 mm) (S1 Table). Stone sizes were categorised into four classes (Table 1). The pottery and amphorae characteristics were recorded within the same analytical units. A total of 3,918 pottery and amphorae sherds (total weight 64,093.3 g) were measured for maximum length, width and thickness (in mm) and weight (in g) (S2 Table). Of these, 2,871 were further classified by the degree of wear (sharp edge, slightly rounded edge, heavily worn sherd). The fragmentation of amphorae was assessed based on maximal length of sherds and divided into four size classes as a result of the relatively uniform qualities of amphorae production (Table 1).

**Table 1:** Stone, amphorae and sherd fragmentation size classes.

Stone size classes	Amphorae size classes	Sherd fragmentation classes
S1 (5-10mm)	A1 (< 40mm)	F1 (SFI < 1)
S2 (10-30mm)	A2 (40 – 100mm)	F2 (SFI = 1–2)
S3 (30-100mm)	A3 (100 – 150mm)	F3 (SFI = 2–4)
S4 (> 100mm)	A4 (> 150mm)	F4 (SFI = 4–8)
		F5 (SFI > 8)

To identify the depositional processes, the sherd fragmentation index (SFI) for pottery other than amphorae was calculated [37, 38]. This index allows comparison of potsherd fragmentation, regardless of the vessel type or size (e.g., fine imported Aco beakers with locally made coarse pottery). SFI is defined by two qualities: weight (in grams, *m*) and thickness of the potsherd (in millimetres, *w*) in the following equation [38]: *SFI* = 5.8 (*m*/w^1.7^). SFI was classified into five fragmentation classes (F1-5) from the relatively small, very fragmented sherds (F1) to large, less fragmented sherds (F5) (Table 1).

Pottery and amphorae were classified and dated based on the established chronology of ceramic finds from Bibracte oppidum [29, 38, 39].

The north-east section of the ditch visible within the complete vertical profile of the trench (Fig 3) was sampled for parasitological, palynological and diatomological analyses, and in four places for micromorphological analysis. Samples were also systematically collected from specific layers during the excavation for plant macroremain and anthracological analyses.

The data from the archaeological excavation have been published in detail [40, 41]. The artefacts and all types of samples collected during the excavation at La Chaume in 2017–2018 are recorded in a database and stored in the European Archaeological Centre in Glux-en-Glenne, France, under intervention numbers 866 (excavation 2017) and 894 (excavation 2018). Soil samples were collected individually according to the requirements of the analytical techniques (as described in detail in the specific sections).

### Radiocarbon dating

Single growing season samples tied to human activity were selected for radiocarbon dating. Samples were retrieved from floated fractions of archaeobotanical sediment samples. The best-preserved charred grains (preferably of one taxon) were selected from strata 2–5 and a charred one-year twig was selected from stratum 1. Pre-treatment (ABA at 60°C, [42]), combustion, graphitisation and radiocarbon measurement were performed at the ETH Zürich AMS radiocarbon laboratory. OxCal and the atmospheric curve with 5-year resolution were used for the radiocarbon calibration (OxCal 4.3 © Christopher Bronk Ramsey 2019, [43]; IntCal13, [44]).

### Sedimentology and micromorphology

The ditch was sampled at four stratigraphic positions (A, B, C and D; Fig 3) and nine thin sections were analysed. The main aim of the analysis was to understand the formation processes. Position A was sampled at the lowest level of the excavations in 2017 to ascertain the presence of Fe/Mn-hydroxy-oxides and micro charcoal, to analyse the state of the organic matter and to evaluate how the recent water saturation may have changed the macroscopic features visible in this position. The *in-situ* sample was 25 cm long, and after impregnation and curing was divided into five thin sections that were divided into four micro-units (A1, bottom, to A4, top). Position B in the central part of the ditch was sampled mainly to ascertain the macroscopically detected soil horizon and to determine its formation processes. The sample from position C included a macroscopically visible white layer (micro-unit C1) and a dark brown soil-like horizon above (micro-unit C2). It was sampled to evaluate the composition of the white layer. Position D represents the bottom of a ditch excavated in 2018 and was sampled to test the hypothesis of the original function of the feature as a water ditch.

*In-situ* sampled blocks of soil were slowly dried and impregnated by Pollylite 2000 resin mixed with acetone in a vacuum chamber. After curing for six weeks, smaller samples were cut off from the blocks and thin-sectioned separately. The thin sections were prepared at the Institute of Geology at the Czech Academy of Sciences in Prague, Czech Republic. The dimensions of the thin sections varied between 3 × 4 cm and 5 × 7 cm. The micromorphological descriptions and interpretations are based on [45–47].

Detailed descriptions and per sample results are published in [40, 41].

### Macroremains and anthracology

Sediment samples (57 samples; 325 litres) for the recovery of seeds and charcoal were systematically collected from individual stratigraphic units (UFs) in three different areas of the ditch: close to the S-E profile, the N-W profile and at the centre of the northern slope of the ditch. To secure recovery of all ecofacts and artefacts, the samples were processed using a combination of flotation, wash-over and wet-sieving [40]. The dried floated fractions were sorted and the plant remains were studied under a stereo microscope (Leica M80 at max 50×). Taxa identification was based on the available literature, and a modern and archaeological reference collection. For charcoal, the refractive surfaces of fragments larger than 2 mm were analysed under a microscope with reflected light (Olympus BX 51 at max 200×). Detailed descriptions and per sample results for carpology and anthracology are published in [41, 48]. Here we present the seed and charcoal results after collating the samples to the wider stratigraphic units corresponding to the main deposition events.

### Palynology

A total of 18 pollen samples, originating from the same positions as the diatom samples (Fig 3), and two modern control surface samples (collected from mosses sampled near the excavation) were analysed. Only the fine fraction of the deposit was used for the sample preparation (volume 0.5 cm^3^), which followed standard acetolysis methods [49] with hydrofluoric acid used repeatedly to dissolve the silica content. At least 250 terrestrial pollen grains were counted in each sample. The determination of pollen grains was based on [49, 50]. Microcharcoals are not displayed against pollen grains due to their low absolute prevailing numbers.

### Diatoms

Diatoms are used worldwide as important bioindicators for water quality. In archaeology, they are used to establish the provenance of archaeological artefacts, for the analysis of archaeological sediments and the reconstruction of local site environments. In general, diatoms are studied in relation to marine, brackish and freshwater environments. However, they are not commonly used to investigate sediments within terrestrial archaeological sites [51]. Diatoms in terrestrial sediments can contribute to the analysis of water management and quality in former wells [52], drainages [53] and ditches [54]. In this study, diatom analysis was initiated after the discovery of waterlogged deposits in the lower part of the ditch.

In total, 28 diatom samples were inspected during pre-screening (presence/absence) followed by identification and enumeration of at least 300 diatom valves (if present) on a permanent slide. Samples were prepared according to [55]. Taxa were identified using [56]. Van Dam indexes from Arrow [57] were used to determine environmental conditions. Saprobity and trophy were determined using the Czech Ecological Quality Index, halobity was determined using the Ec-index and aerotolerance was determined using the Aer/Aq Index [58].

### Parasitology

In total, 33 samples were collected for parasitological analysis. Of those, 30 samples originated from the section (Fig 3) and three from the bottom of the ditch (G1-3; cf. Fig 4). The samples were collected by trowel in clean single-use 60 ml universal sample containers (Greiner Bio-One). The samples were then handled under clean conditions at the Department of Zoology, University of Oxford, United Kingdom (described in [28]). A sub-sample (5–10 g) was re-hydrated and disaggregated overnight in 20 ml of ultra-pure water (Sigma-Aldrich). Two rounds of microscopy were carried out (Nikon Eclipse E400 with Nikon 20×/0.25 Ph1 DL and 40×/0.65 Ph2 DL objectives). Prior to microscopic analysis, only agitation before pipetting was carried out to stop denser material settling. A QImaging MP5.0 RTV camera was used with QImaging QCapture Pro to record any suspected eggs and these images were assessed against reference images before the final count number was confirmed.

## Results

### Geophysics

**Fig 5:**
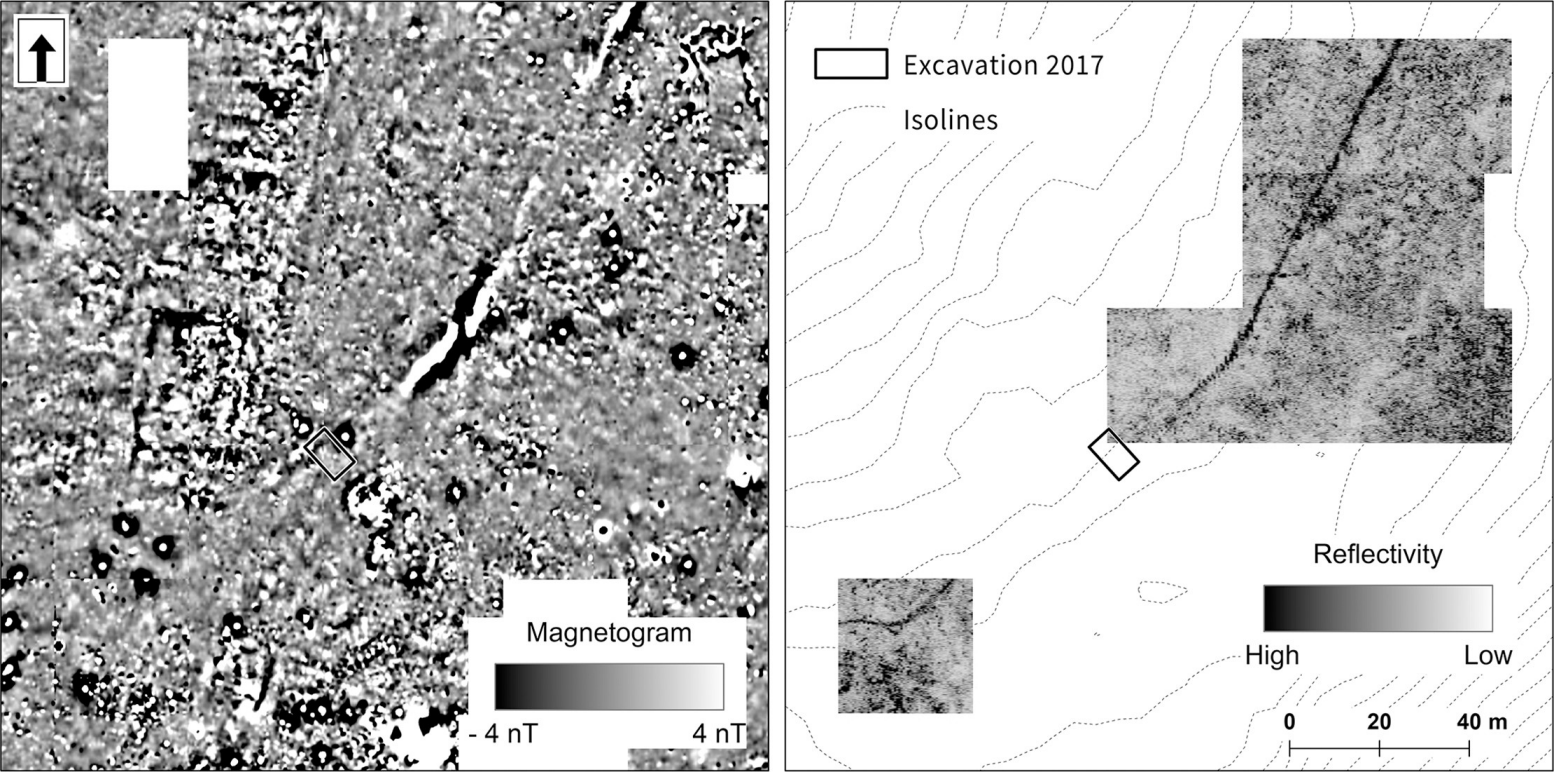
Bibracte/Mont Beuvray—La Chaume. Results of magnetometer (left) and GPR (right) surveys. Ditch UF 180 is visible as a clear line that runs in the NE-SW direction.

Magnetometer surveys at La Chaume, the central part of the summit plateau of Mont Beuvray covering an area of nearly 25 ha, detected an almost 270 m long and 3–4 m wide linear structure stretching between the fortifications at La Terrasse and Le Porrey (Fig 5) and continuing beyond the surveyed area. This structure was invisible in the topography and LiDAR data. The structure runs in the SW-NE direction following contour lines with an altitude of 811–812 m. We interpreted it as a ditch separating the highest part of La Chaume from its surroundings.

In the magnetic survey, the ditch was only slightly visible in some parts due to the low magnetic values (0.5–2 nT). However, high magnetic values (up to 100 nT) were detected in two sections approximately 40 m and 50 m in length, respectively. This indicated that the ditch filling was composed of diverse material in these sections.

The ditch was visible in georadar data as a homogeneous linear structure. We expected a significant representation of stones in the ditch filling. The original estimation assumed a depth of more than 3 m. The subsequent excavation showed that the ditch was shallower. The lower part of the ditch, together with its immediate surroundings, was saturated with water, which greatly influenced the results of the georadar survey.

### Archaeology

Based on the geophysical survey, a trench was cut across the newly detected linear feature. The excavations unearthed a ditch 4.5 m wide at the top and 1.8 m deep. The bottom was located 2.5 m beneath the modern walking horizon. The complete transverse section of the ditch was documented in two longitudinal sections of the trench (Figs 2–4).

Based on the combination of archaeological stratigraphy, sedimentology and micromorphology, the individual layers were grouped into five strata.

Dating based on artefacts (mainly pottery).

Stratum 1 was composed of organic black sandy loam covered by turf. This stratum was overlying the infill of the ditch and was disturbed by numerous later cuts. It had an inhomogeneous context with a mixture of finds. These included contemporary refuse (e.g., Orangina bottle), Modern period (e.g., 19^th^ century military button), Post-Medieval and Medieval, alongside artefacts from the 1^st^-4^th^ century AD (Gallo-Roman period) and the 1^st^ century BC (La Tène occupation of the oppidum).

The deposits in strata 2 to 5 represented the infill of the ditch (Fig 3; Table 2). The majority of the ceramic and metal artefacts were dated to the 1^st^ century BC. Medieval finds rarely occurred in stratum 2 [40, 41].

**Table 2:** Bibracte/Mont Beuvray—La Chaume (ditch UF180). Description and chronology of the infill.

Strata	Context	Description	Dating
1	100, 109	Organic black sandy loam under turf, overlying the infill of the ditch	Iron Age—La Tène D2b, Gallo-Roman period, Medieval, Post-Medieval, Modern
2	110, 116, 174, 159, 169, 118; 118, 204, 307, 217	Upper infill of the ditch	La Tène D2b - early Augustan period (50–20 BC) and earlier LT D1 material (120–50 BC)
3	128, 182; 245 = 254	Middle infill of the ditch	La Tène D2b (50–30 BC)
4	177, 178, 183; 263, 259, 281, 262	Lower infill of the ditch	La Tène D2b (50–30 BC) and older LT D1 material (120–50 BC)
5	275	Lowest infill of the ditch	La Tène D2b (50–30 BC)

In total, 4,220 amphorae fragments (minimum number of individuals, MNI 169; weight 191.5 kg) and 2,158 pottery fragments (MNI 276) were obtained [41]. The amphorae and pottery assemblages from a more thorough excavation of strata 2 to 5 undertaken in 2018 (2,887 amphorae sherds, 56.2 kg; 988 pottery sherds, 6.9 kg) were subjected to more detailed analysis.

**Fig 6:**
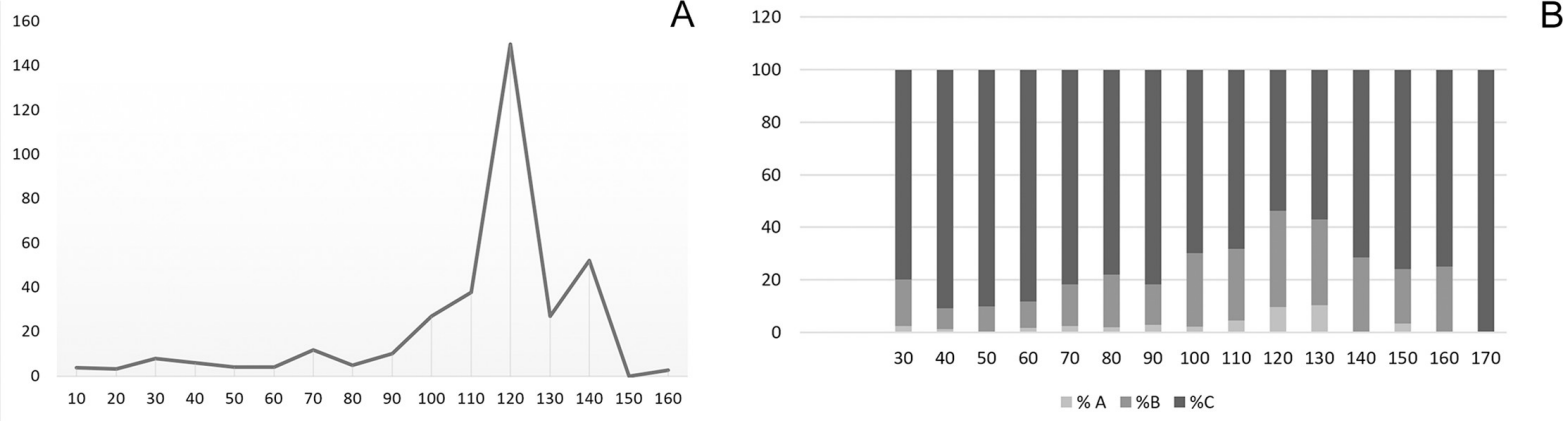
Bibracte/Mont Beuvray—La Chaume (ditch UF180). (A) Density of amphorae fragments (X-axis–depth; Y-axis–density in g/l). (B) Proportional representation of pottery sherd wear at various depths of the trench (cm); A–little wear, B–medium wear; C–heavily worn; for number of sherds cf. S2 Table.

**Fig 7:**
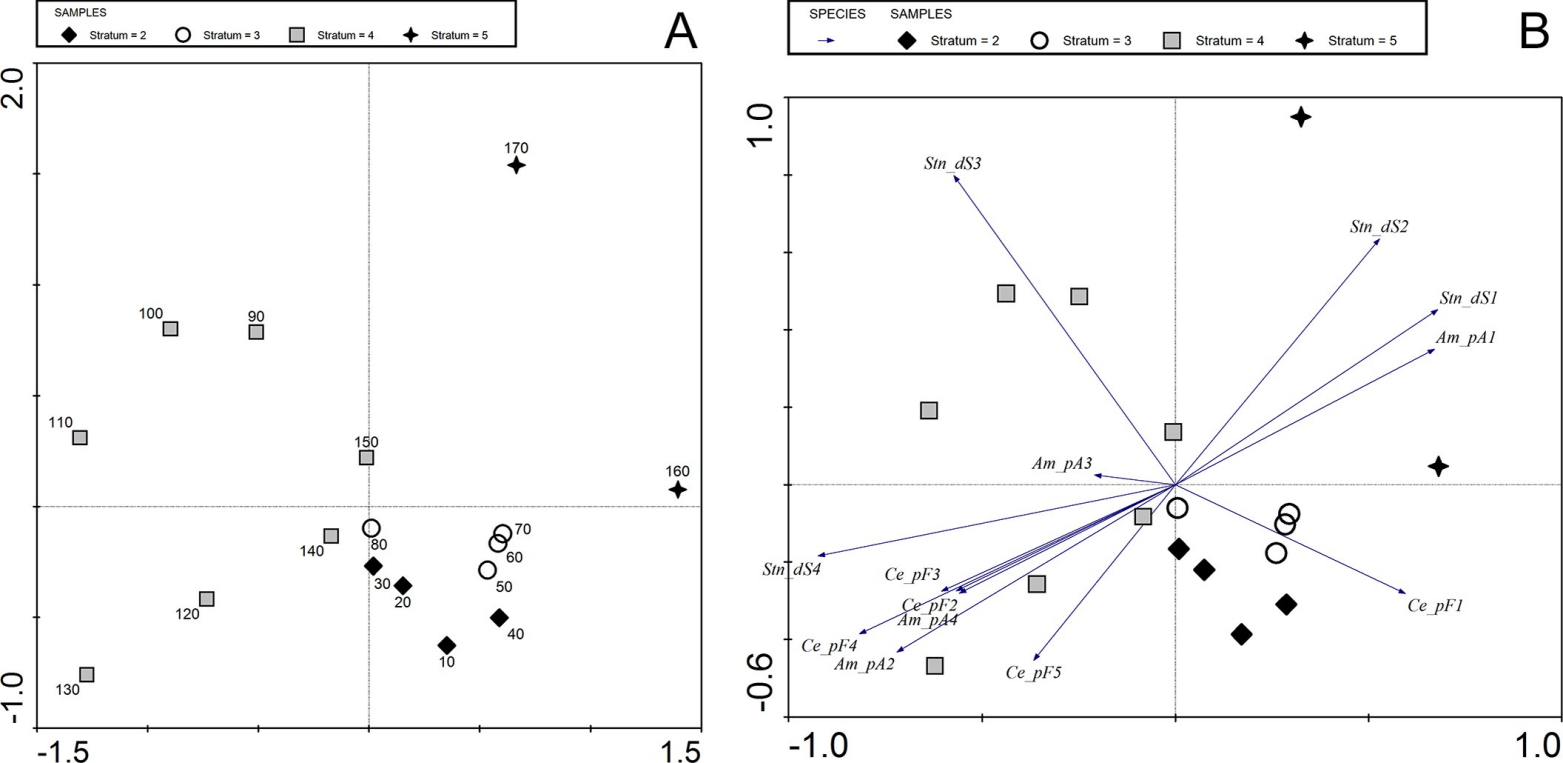
Bibracte/Mont Beuvray—La Chaume (ditch UF180). Bibracte/Mont Beuvray—La Chaume (ditch UF180). Principal component analysis of fragmentation data on ceramic and amphorae finds and stone size. (A) Sample plot with numbers denoting the sample depth (see Fig 4). (B) Biplot of sample and environmental data (Ce = pottery, Am = amphorae, St = stone, d = size, p = fragmentation; for further legend see Table 1).

The density of amphorae sherds in the ditch infill was the highest in stratum 4. The amphorae fragments in the higher strata 2 and 3 were smaller and showed a higher degree of wear (on average consisting of 86% heavily worn sherds) than in the lower strata 4 and 5 (on average 66% heavily worn sherds) (Figs 6 and 7).

Similar to the amphora fragments, the most fragmented pottery sherds (F1) were best represented in the upper strata 2 and 3. Stratum 4 was characterized by a high proportion of less fragmented pottery (F3-5; Fig 7).

The observed differences between strata 2 and 3, and strata 4 and 5 can be attributed to different depositional histories [59, 37, 60]. Stratum 5 contained a small amount of highly fragmented amphorae and pottery (none at the bottom), which represents the period over which the ditch was gradually being filled-in but kept clean from waste. The rapid, probably intentional, filling of the lower part of the ditch (stratum 4) was likely carried out with material from an unknown, short-time refuse area, which allowed relatively large fragments originating from a plethora of different vessels to be deposited in one stratum (cf. [59]). In the entire assemblage of 3,875 sherds only twenty times two sherds (not glued together) were identified as part of the same ceramic object, and only one 2-sherd object and one 13-sherd object were detected. It is unlikely that those large sherds came from the immediate vicinity (dump area or settlement next to the ditch) because the number of sherds from the same vessel would be higher [20, 37]. The lower strata of the ditch (4 and 5) also had a higher density of stone components, with the highest share of large stones found in stratum 4 (Fig 7).

Regarding the taphonomic origin of the material in strata 2 and 3, the small size and weathering of the edges of the sherds indicate that they most probably remained on the surface of the settlement or in a refuse area for a long period of time or were repeatedly moved between deposits [37].

### Radiocarbon dating

The results of the radiocarbon analysis are summarised in Table 3.

**Table 3:** Bibracte/Mont Beuvray—La Chaume (ditch UF180). La Tène ^14^C dates, vague-prior calibration.

Strata	Site sample code	Laboratory sample code	C14 age BP±1σ	Unmodelled date	δC13‰±1σ	mg C	C/N ratio	Species	Description
1	B2017.12.109.107	ETH-92294	875±22	1048–1220 calAD	-29,0±1	1,00	75,76	*Corylus avellana*	wood charcoal 1 year twig
2	B2017.12.116.146	ETH-92296	2132±23	347–61 calBC	-24,0±1	0,99	24,50	*Triticum dicoccum*	cereal grain
2	B2017.12.110.113	ETH-92295	2105±22	191–54 calBC	-25,3±1	0,99	23,10	*Triticum dicoccum*	cereal grain
3	B2017.12.128.148	ETH-92297	2096±22	179–50 calBC	-24,1±1	0,99	22,62	*Triticum dicoccum*	cereal grain
4	B2017.12.178.168	ETH-92298	2093±23	179–48 calBC	-26,7±1	1,00	28,45	*Hordeum vulgare*	cereal grain
5	B2017.12.275.205	ETH-92301	2052±23	163 calBC—4 calAD	-26,2±1	0,99	18,48	*Triticum spelta/ aestivum*	cereal grain

The site sample code allows the identification of the sample within the catalogues of the excavation. Based on the analysed La Tène samples, only a wheat grain (ETH-92301) from bottom layer 275 could represent settlement activity in 50–30 BC or later. The probability of the unmodelled date is 95.4%.

**Fig 8:**
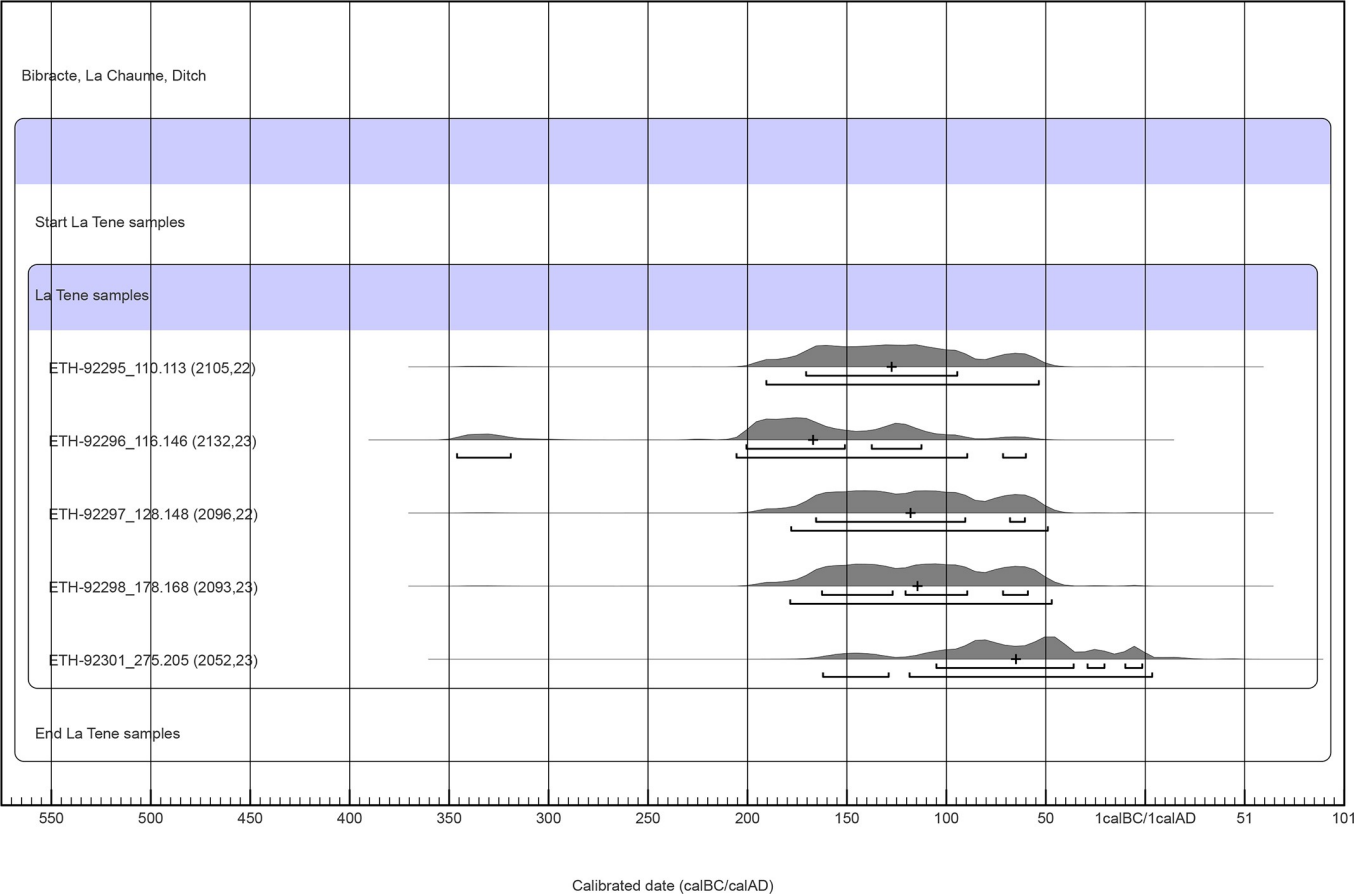
Bibracte, ditch at La Chaume. La Tène ^14^C dates, vague-prior calibration. The numbers after the laboratory code denote the archaeological layer and the archaeobotanical sample. According to the vague-prior calibration, only ETH-92301 from the bottom layer (UF275) could represent an archaeological event from 50–30 BC or later. The one and two sigma ranges with medians are shown in the figure.

In accordance with the site habitation history, the one-year *Corylus avellana* twig from stratum 1 is of High Medieval age (Table 3). Regarding the two La Tène habitation periods visible in the pottery (120–50 BC and 50–30 BC; Table 2), the remaining radiocarbon dates, with the exception of one, either predated 50 BC or were dated around that date (Table 3, Fig 8). Only the grain fragment from the layer at the bottom of the ditch (UF275) could represent agricultural activity from 50 cal BC onwards (Figs 3 and 8; Table 3). This would be in agreement with La Tène D2b pottery in this layer (50–30 BC; Table 2). However, the calibrated range was too large to exclusively date the grain fragment to the second half of the 1^st^ century cal BC (Fig 8) because archaeological events from the 70s, 60s, and 50s BC could also result in radiocarbon dates ending around 1 cal AD (IntCal13: [44]).

**Fig 9:**
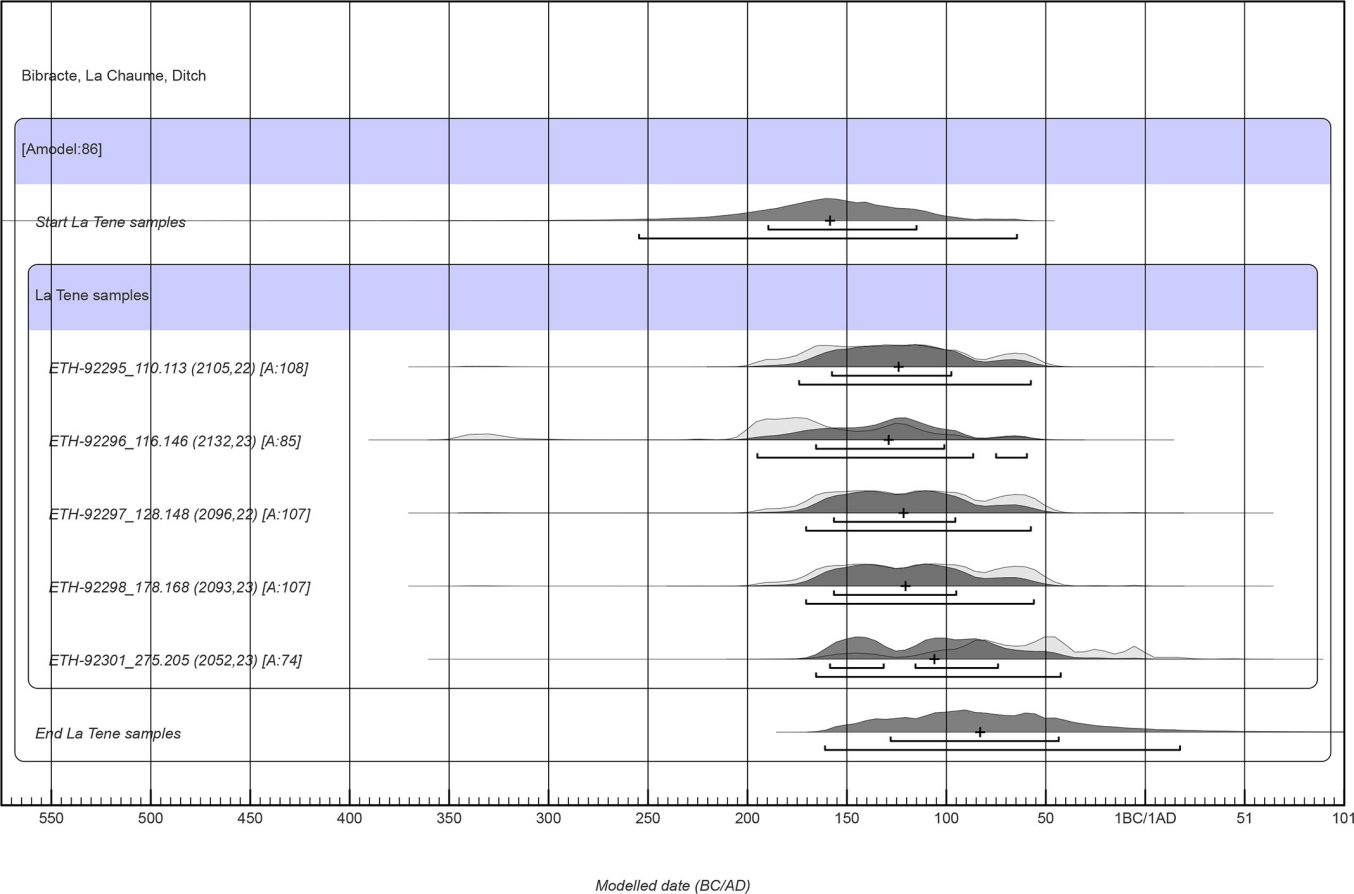
Bibracte, ditch at La Chaume. La Tène ^14^C dates, single bounded phase model. The median for the start boundary is 159 cal BC and the median for the end boundary is 84 cal BC. The one and two sigma ranges with medians are shown in the figure.

Owing to the redeposited nature of sediment in the ditch and the small-size sample mobility, a uniform phase model was chosen for the calibration of the measured ^14^C determinations. The start and end boundaries of the uniform phase indicate the start and end of agricultural activity represented by the dated cereal grains, and thus, the beginning and end of La Tène habitation. To express the beginning and the end, the medians of the start and end boundaries can be used, which are 159 cal BC and 84 cal BC, respectively (Fig 9). However, these values do not necessarily represent accurate estimates. Our analyses using the simulated radiocarbon dates from around 120–30 BC showed that the medians of the boundaries may well under- or over-represent true values by several years or decades.

### Sedimentology and micromorphology

The infill of the ditch (Fig 3) was composed of moderately sorted or unsorted material with stone accumulations occurring only in specific parts. In general, five main lithological facies covering separate lithological units were distinguished macroscopically in the infill of the ditch (Table 4). The transitions between the layers (cf. Fig 3) were sharp or abrupt.

**Table 4:** Bibracte/Mont Beuvray—La Chaume (ditch UF180). Sedimentological description of the main lithological facies.

Stratigraphic units (UF)	Description
110, 115, 118, 159, 169, 178, 183	Unsorted sandy loam containing angular stone fragments (few cm in diameter) with light beige colour formed the bottom part of the infill
180	Infill contained a black coloured tough band, macroscopically suggesting the presence of Mn/Fe hydroxy-oxides
178	Light brown unsorted sandy loam containing angular stone fragments (few cm in diameter)
109, 128	Dark-coloured organic rich deposits were detected as relatively thick layers (few cm), these were silty to sandy loam and rich in stone fragments
116	Silty to sandy loam and rich in stone fragments. For UF116, a thin white band with sharp transitions below and above was detected.
100	Grey black silty loam containing angular stone fragments (few cm in diameter); these deposits were highly bioturbated with a number of roots and earthworm features.

**Fig 10:**
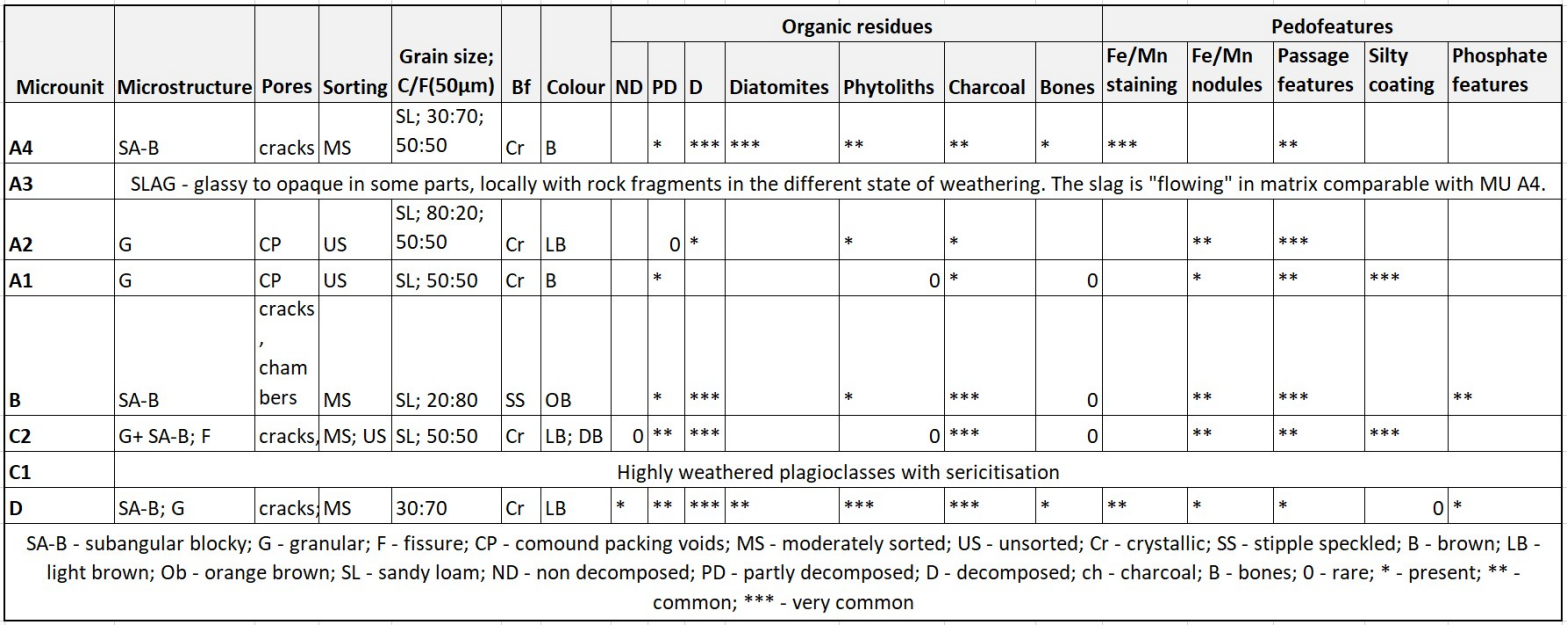
Bibracte/Mont Beuvray—La Chaume (ditch UF180). Micromorphological description of individual micro-units. SA-B—subangular blocky; G—granular; F—fissure; CP—compound packing voids; MS—moderately sorted; US—unsorted; Cr—crystallic; SS—stipple speckled; B—brown; LB—light brown; Ob—orange brown; SL—sandy loam; ND—non decomposed; PD—partly decomposed; D—decomposed; ch—charcoal; B—bones; 0—rare; *—present; **—common; ***—very common.

Micromorphological analysis of samples from positions A-D included four strata (2–5). The detailed description of individual micro-units is presented in Fig 10.

Position D (stratum 5) was composed of a fine-grained matrix. While the number of diatoms seemed to be low, micro-charcoal, fine grained fragmented organic matter and phytoliths were common. There was no lamination suggesting flowing water and the material most probably accumulated into standing water over an extended time period (weeks, years). The bioturbation of this material is clearly visible. The more intensive accumulation of the material into the ditch began with a black layer/lens (possibly slag).

Position A (stratum 4) was composed of a similar matrix as position D. However, it was rich in diatoms, micro-charcoal and bone fragments (mostly micro-units A3 and A4). Bioturbation was fairly common (mostly micro-unit A2). The black colour was due to Fe/Mn staining, and the presence of organic matter and slag fragments (mostly micro-units A3 and A4). Position B (stratum 3) was a well-developed, anthropogenically-influenced and homogeneous soil A-horizon rich in phosphates (micro-unit B). A soil A-horizon was detected within position C (stratum 2) (micro-unit C2). Micro-unit C1, located below C2 and macroscopically observed as a white layer, was composed of highly weathered plagioclasses with sericitisation. The bottom part of the plagioclase layer contained thin bands of Fe-hydroxyoxides. The composition of micro-unit C1 suggested an anthropogenic origin (possibly mortar).

### Archaeobotany

The 56 samples that were evaluated included an assemblage of charred (NISP 225) and uncharred (NISP 699) plant remains (details in [48]). The expected prehistoric or medieval dating of the majority of the charred remains was supported by their direct radiocarbon dating (Table 3). The uncharred remains of taxa occurring in the area today were all considered modern contaminants that were introduced into the archaeological strata via (bio)turbation. They were evaluated based on numbers only and were used as a taphonomic proxy (Table 5).

**Table 5:** Bibracte—La Chaume, the different strata of the fill in ditch UF180 and the associated plant macroremains.

Stratum	6	5	4	3	2	1	0	Total	Ubiquity
Number of samples	1	8	15	2	19	6	1	57	
Volume [litres]	1	26	101	6	111	47	6	325	
**Grain**									
*Panicum miliaceum*	--	3	4	1	8	3	--	19	19.6
*Setaria italica*	--	1	1	--	3	--	--	5	7.1
*Hordeum vulgare* var. *vulgare*	--	--	29	3	11	--	--	43	32.1
*Triticum spelta*	--	1	6	--	3	--	--	10	12.5
*Triticum dicoccum*	--	2	5	1	5	--	--	13	16.1
*Triticum aestivum* s.l.	--	2	--	--	--	--	--	2	1.8
*Triticum monococcum*	--	--	2	--	4	--	--	6	7.1
*Triticum / Hordeum*	--	4	13	2	12	1	--	32	37.5
*Avena* sp.	--	--	--	--	1	7	--	8	8.9
*Secale cereale*	--	--	--	--	2	1	--	3	5.4
**Chaff**									
*Triticum* spp. (sf)	--	3	1	--	2	--	--	6	7.1
*Secale cereale* (ri)	--	--	--	--	--	1	--	1	1.8
*Cerealia* (straw, rhizomes)	--	--	--	--	--	1	--	1	1.8
**Legumes**									
*Lens culinaris*	--	--	1	--	1	1	--	3	5.4
*Vicia* cf. *faba*	--	--	1	--	--	--	--	1	1.8
Leguminosae Sativae indet.	--	2	2	--	5	--	--	9	14.3
**Fruits**									0.0
*Prunus* cf. *persica* (fr.)	--	1	--	--	--	--	--	1	1.8
*Corylus avellana*	--	5	3	--	5	4	--	17	16.1
*Prunus* cf. *spinosa*	--	--	1	--	--	--	--	1	1.8
**Wild plants**									
**Arable weeds**									
*Brassica/Sinapis*	--	--	1	--	--	--	--	1	1.8
*Bromus secalinus/B*. *arvensis*	--	--	2	--	--	--	--	2	1.8
*Centaurea cyanus*	--	--	--	--	1	--	--	1	1.8
*Galium* sp.	--	--	--	--	1	--	--	1	1.8
*Polygonum persicaria*	--	--	1	--	3	--	--	4	7.1
*Rumex acetosella*	--	--	--	--	--	2	--	2	3.6
*Vicia tetrasperma/V*. *hirsuta*	--	--	1	--	--	--	--	1	1.8
*Chenopodium album* aggr.	--	--	--	--	1	--	--	1	1.8
**Grassland or field**									
*Poa annua*/*Holcus lanatus*	--	--	--	--	--	3	--	3	1.8
*Poa* spp.	--	--	--	--	--	2	--	2	3.6
**Wet grasslands**									
*Rumex acetosa/R*. *crispus/R*. *conglomeratus*	--	--	1	--	3	--	--	4	8.9
*Rumex maritimus/R*. *palustris*	--	--	--	--	--	1	--	1	1.8
*Stellaria graminea*	--	--	--	--	--	1	--	1	1.8
*Carex* sp.	--	--	--	--	1	--	--	1	1.8
**Forest and edges**									
*Abies* sp., *Picea* sp. (needles)	--	--	--	--	2	3	--	5	5.4
Not determined	--	2	7	--	4	1	--	14	17.9
Charred (total)	0	26	82	7	78	32	0	***225***	
Uncharred (total)	0	0	0	0	49	55	595	***699***	

The numbers refer to MNI.

The samples were collated into wider stratigraphic units (strata 1–5), a bedrock stratum (6) and the plough-soil horizon (0).

All samples were ‘grain/chaff-poor’, but the species of crops and wild plants were the same as those described in previous studies from Bibracte [61–63]. The lower strata (3–5) contained taxa characteristic of the protohistoric period at the site, e.g., combination of glume wheats (*Triticum dicoccum*, *T*. *spelta*, *T*. *monococcum*) and barley (*Hordeum vulgare*). In the upper strata (1–2) these were mixed with species more common in medieval times, such as millet (*Panicum miliaceum*), oat (*Avena* sp.) and rye (*Secale cereale*).

All recovered plant remains were poorly preserved, distorted and/or fragmented. Less than half of the cereal grains could be determined to species level. The highest fragmentation rate and poorest preservation occurred in stratum 2. The plant remains in the lower strata 5 and 4 were slightly better preserved.

The bedrock (stratum 6) did not contain any seeds, charred or uncharred. Strata 5 and 4 contained only charred plant remains with cereal taxa characteristic of the La Tène period at the site. Stratum 5 contained *Panicum miliaceum*, *Setaria italica*, *Triticum spelta*, *T*. *dicoccum* and *T*. *aestivum*/*T*. *durum*. Stratum 4 also included *T*. *monococcum* and *Hordeum vulgare* var. *vulgare*. The identified legumes were *Lens culinaris* and *Vicia faba*. The wild plants seeds that could be identified were mostly arable weeds or wet grassland taxa. Stratum 3 was the thinnest stratum, and thus, the least sampled. It was also the poorest, containing only seven charred grains (millet, barley, and emmer). Stratum 2 contained an equal number of charred and uncharred remains. Charred protohistoric glume wheats occurred alongside *Secale cereale* and *Avena* sp. characteristic of the Medieval period and charred needles of *Picea abies* introduced to Bibracte only in Modern times. Stratum 1 had fewer charred than uncharred seeds. Charred grain and chaff represent a typical medieval combination of millet, rye and oat. The charred seeds of four grassland plants and one arable weed occurred alongside the charred rhizome of a grass/cereal. Similar to stratum 2, charred conifer needles were present. Only uncharred plant remains were present within the plough-soil horizon (0), with seeds representing the modern soil seed bank.

### Charcoal

Beech (*Fagus sylvatica*) represented over 50% and oak (*Quercus* sp.) 26% of the charcoal assemblage (NISP 1915, weight of determined specimens 85.65 g, total charcoal weight 161.5 g). Both were present in a similar number of samples (Table 6).

**Table 6:** Bibracte—La Chaume, the different strata of the fill in ditch UF180. Weight of taxa determined in wood charcoal (in g).

Stratum	5	4	3	2	1	WISP [g]	NISP	Ubiquity
*Fagus sylvatica*	6.13	15.61	2.07	25.88	1.44	51.12	1104	41
*Quercus* sp.	3.15	6.66	3.27	5.57	1.86	20.51	522	42
*Corylus avellana*	0.57	0.62	0.33	0.62	0.61	2.76	77	26
*Cytisus scoparius*	0.05	0.79	0.20	0.75	0.29	2.07	44	21
*Alnus* sp.	0.17	0.79	0.35	0.38	--	1.70	37	21
*Fraxinus* sp.	0.31	0.11	--	3.61	0.07	4.09	34	16
*Pomoideae*	0.04	0.33	0.18	0.13	0.06	0.74	18	14
*Betula* sp.	0.03	0.41	0.21	0.31	--	0.96	21	11
*Betula/Alnus*	0.02	0.04	--	--	--	0.06	3	3
*Quercus/Castanea*	0.02	0.42	--	0.42	0.04	0.90	32	14
*Populus/Salix*	0.02	0.30	--	0.17	--	0.49	18	5
*Populus* sp.	--	0.03	--	--	--	0.03	2	1
*Acer* sp.	--	0.11	--	--	--	0.11	1	1
*Sambucus* sp.	--	--	--	0.06	--	0.06	1	1
*Carpinus betulus*	--	--	--	0.04	--	0.04	1	1
residue	9.70	22.54	9.95	25.14	8.49	75.98	--	--
**Total determined**	10.52	26.20	6.61	37.95	4.36	85.65	--	--

WISP: weight [g] of identified specimens; NISP: number of identified specimens; Ubiquity: number of samples in which the taxa occurred.

The third most commonly found species was hazel (*Corylus avellana*). Species from open woodlands (cf. *Cytisus scoparius*, *Betula* sp.) and wet soils (*Alnus* sp., *Populus* sp., *Populus*/*Salix*) were moderately common. Tentatively identified chestnut (*Castanea/Quercus*) and *Pomoidae* (Table 7) were common. Hornbeam (*Carpinus betulus*), maple (*Acer* sp.) and elderberry (*Sambucus* sp.) were rare.

**Table 7:** Functional trait categories of diatom lifestyle.

Category	Diatom lifestyle
FT1	planktonic taxa (centric diatoms)
FT2	typically periphytic taxa adhering to the surface
FT3	facultatively benthic/planktonic araphid taxa
FT4	facultatively benthic/planktonic taxa with raphe
FT5	actively moving epipelic taxa

**Fig 11:**
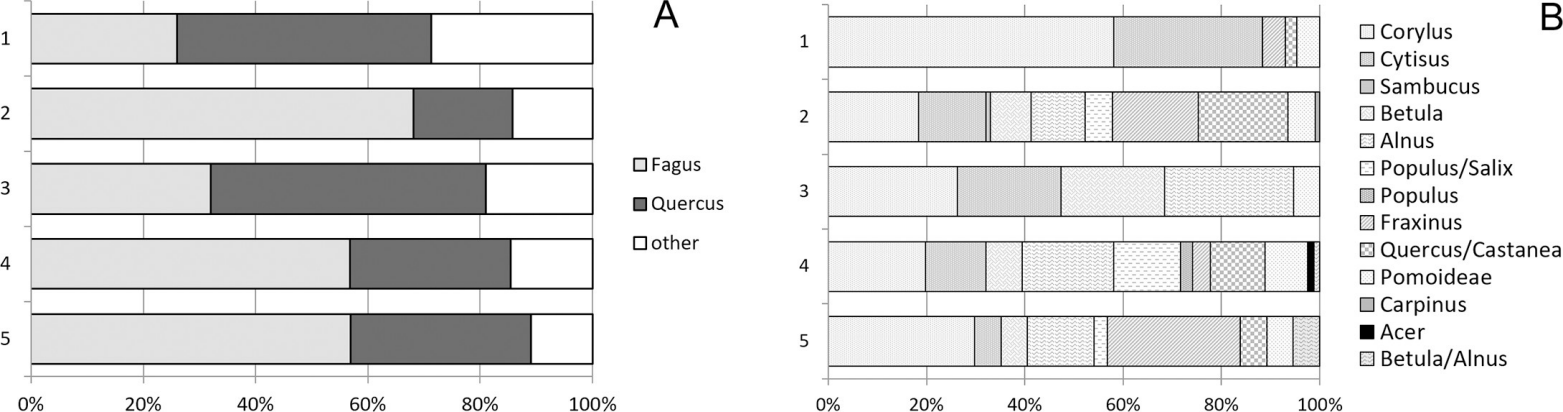
Bibracte, La Chaume–ditch (UF 180). (A) Charcoal NISP values of beech, oak and other taxa per stratum. (B) Percentages of individual taxa within « other » categories (based on NISP).

The charcoal and the taxa composition varied across the strata (Fig 11). Stratum 5 had less charcoal (9.7 g) than stratum 4 (22.5 g), but both comprised of almost 60% *Fagus* and over 20% *Quercus*. Stratum 5 had more *Fraxinus*, whereas stratum 4 had more charcoal from trees growing in wet soils like *Alnus* and *Populus*/*Salix*. The upper ditch fills (strata 3 and 2) were also different, although this could be due to a low number of samples in stratum 3 (two samples) compared to stratum 2 (17 samples). Stratum 3 contained slightly more *Quercus* (3.27 g) than *Fagus* (2.07 g) as well as the largest proportion of *Alnus* in the assemblage. Stratum 2 contained the highest amount of charcoal overall (over 700 fragments, 37.9 g), dominated by *Fagus* with abundant tentatively identified chestnut *(Castanea*/*Quercus)*. For the first time *Sambucus* and *Carpinus* were identified. The overlying stratum 1, despite consisting of over 100 fragments, contained the most common taxa and ash, chestnut/oak and *Pomoidae*.

A comparison of our assemblage with earlier data from the site [64] revealed two main differences. Contrary to reported data, our assemblage contained more *Fagus* than *Quercus*. Second, the spectra of trees and shrubs did not cover all the taxa recorded in the charcoal or waterlogged wood. Previous reports on charcoal also identified *Prunus* cf. *padus*/*avium*, *Alnus glutinosa*, *Hedera helix*, *Juglans regia*, *Abies alba* and *Tilia* sp. Uncharred wood also contained *Juniperus communis*, *Ulmus* sp., *Lonicera* sp. and *Ilex* sp.

### Palynology

**Fig 12:**
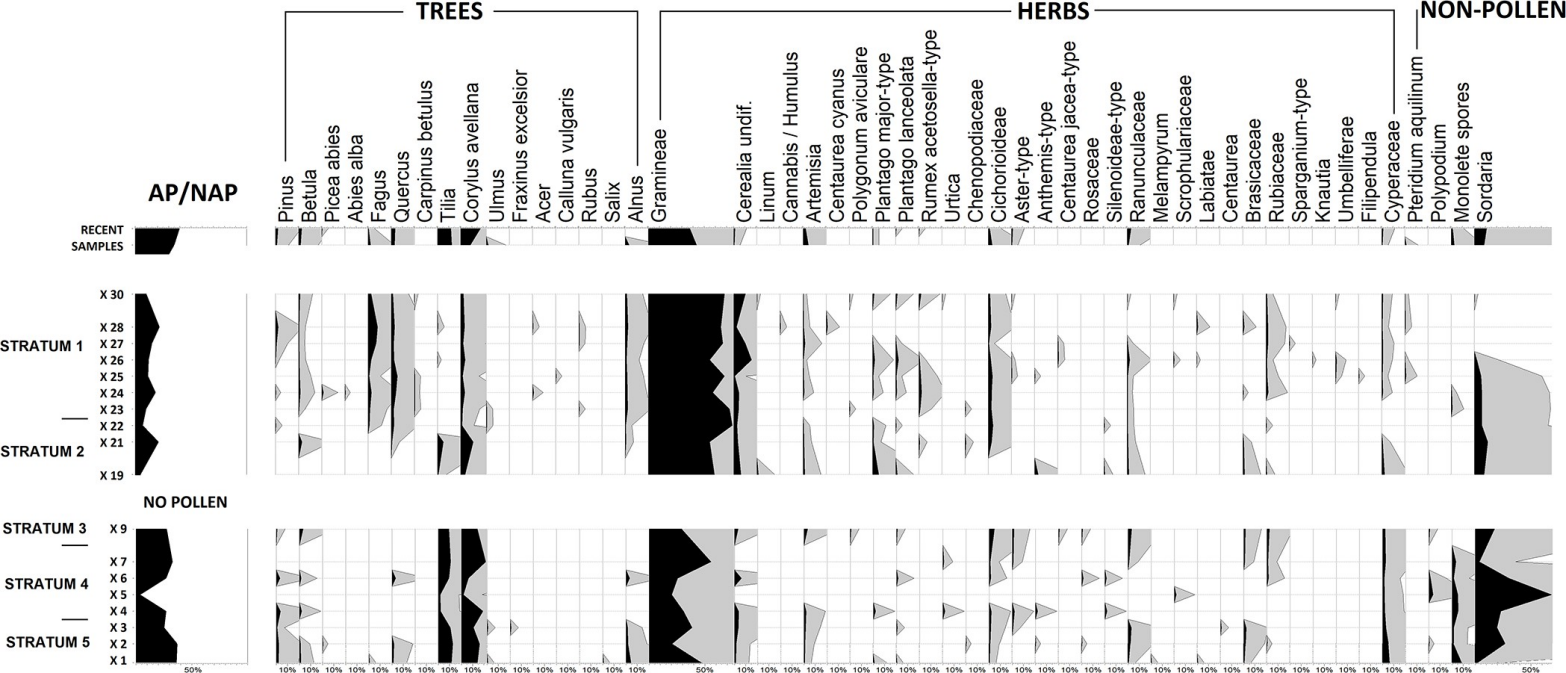
Bibracte/Mont Beuvray—La Chaume (ditch UF180). Percentage pollen diagram of selected taxa.

Pollen grains were only preserved in strata 5, 4, 2 (sparsely) and 1 (Fig 12). The grains were often damaged or corroded and did not allow clear identification. Microcharcoals were the most abundant particles, with the majority consisting of grasses. Wood microcharcoals were rare, but were most abundant in strata 2 and 1.

Strata 5 and 4 had a ratio of arboreal (AP) to non-arboreal pollen (NAP) of around 35%. The woodland was mainly composed of *Corylus* and *Tilia*; whereas *Betula*, *Pinus* and *Quercus* were less frequent. The high abundance of *Tilia* was interesting because it is rarely found in regional pollen records [65]. The herbal spectrum was dominated by grass pollen. The occurrence of cereal pollen was low (below 5%) and only a single *Linum* grain was observed representing other crops. There was a low frequency of *Rumex acetosella*-type and *Plantago lanceolata*, which are indicators of grazed and trampled sites. The presence of fungal ascospores of *Sordaria* indicate that strata 5 and 4 were formed by the accumulation and decomposition of organic material, and the monolete spores of mosses and ferns indicate humid conditions.

Stratum 2 (samples X-19 and X-21) had an AP/NAP ratio of around 20% (lower than strata 4 and 5), but its composition of woodland species was similar.

Stratum 1 had a different woodland composition compared with strata 2, 4 and 5. The abundance of *Fagus*, *Quercus* and *Corylus* corresponded with Medieval and present-day vegetation. Other trees such as *Betula* and *Pinus* were less common. Interestingly, *Alnus* was present, although the site was outside of alluvium. The abundance of grass pollens in stratum 1 was over 60%, which is higher than that in the lower strata 4 and 5. Cerealia pollen peaked in stratum 1 reaching up to 20%. *Cannabis* pollen was significant in the region during the Medieval period. Indicators of trampling (e. g., *Plantago major*) and open habitats (e. g. *Cichorioideae*) were common. *Pteridium aquilinum* indicated pauperisation and disturbed woodland.

The contemporary control moss samples had an AP/NAP ratio of 45% (higher than archaeological ones), with a rising abundance of *Corylus* and *Tilia*. There was a slight decline in grass pollen in the herbal part of the pollen sum, but otherwise the species were as abundant as in strata 1 and 2.

### Diatoms

In total, 49 diatom taxa were found (45 species of pennate diatoms and four species of centric diatoms) (S3 Table). Species richness ranged from 3 to 20 diatom taxa per sample. The highest species richness was calculated for samples X1, X2, X6 and X7 (more than 17 taxa) and the lowest for samples X13, X14 and X21 (3 taxa in each of these samples). The overall diatom diversity was fairly low. However, the samples contained sufficient numbers of bioindicators to deduce the ecological conditions of the period during the formation of the ditch infill.

The abundance of diatoms in samples X1-X9, X11, X19 and X24 was sufficient to count 300 individuals per sample on a permanent slide. The quantified samples from strata 5 and 4 (X1-X9) contained 13–20 species per sample, and those from strata 3 and 2 (X11, X19 and X24) contained 6–16 species per sample. The remaining samples contained only 0–3 species per sample as well as only a few individuals (0–36).

The species composition of the diatom samples indicated a good water quality corresponding to β-mesosaprobity and mesotrophy. The values of the Czech Ecological Quality Index range from 2 (strata 4, 5) to 1 (strata 1–3), which shows that water quality tended to improve from the bottom of the ditch to the surface. Samples also contained indicators of clean water (oligosaprobity and oligotrophy), such as different species of the genus *Eunotia* (e.g., *E*. *bilunaris*) and *Pinnularia* (e.g., *P*. *sinistra*, *P*. *viridiformis*), *Fragilaria rumpens* and in small counts *Fragilaria virescens*, *Gomphonema coronatum*, *G*. *subclavatum* and *Tabellaria* sp. Taxa indicating poor water quality (α-mesosaprobity–polysaprobity, eutrophy–hypertrophy) appeared only in small counts (e.g., species *Hippodonta capitata*, *Mayamaea atomus*, *Surirella angusta* and representatives of the genera *Craticula* and *Nitzschia*). The EC Index of halobity fluctuated around 2 (for all strata), which showed that the species composition corresponded with medium halobity/conductivity with the absence of typically halobiont taxa. The presence of several species of the genera *Eunotia* and *Pinnlaria* and of the species *Gomphonema coronatum* indicates a slightly acidic pH (acid bedrock).

The species composition of the X1-X6 samples (stratum 5 and lower part of stratum 4) indicated standing water (e.g., planktonic centric diatom *Puncticulata* in samples X1 and X6). Sample X7 (middle part of stratum 4) represented a threshold between an aquatic and a terrestrial environment, indicated by the large numbers of aerotolerant taxa *Hantzschia amphioxys* and *Pinnularia borealis*. These two aerotolerant taxa also dominated the remaining samples (from X8 to X28), indicating a terrestrial environment.

The samples mainly contained pennate diatoms and few individuals from planktonic taxa (centric diatoms). Pennate diatoms were either exclusively benthic taxa (living in the river/bottom of standing water) or facultatively benthic/planktonic taxa (i.e., alternating between a planktonic and benthic lifestyle during the life cycle). Some of the pennate diatom genera with a raphe (raphid genera) are able to move actively, while araphid genera (without a raphe) move only passively. To describe these particular diatom taxa life styles, five functional trait categories (FT) were used after [66] (Table 7).

**Fig 13:**
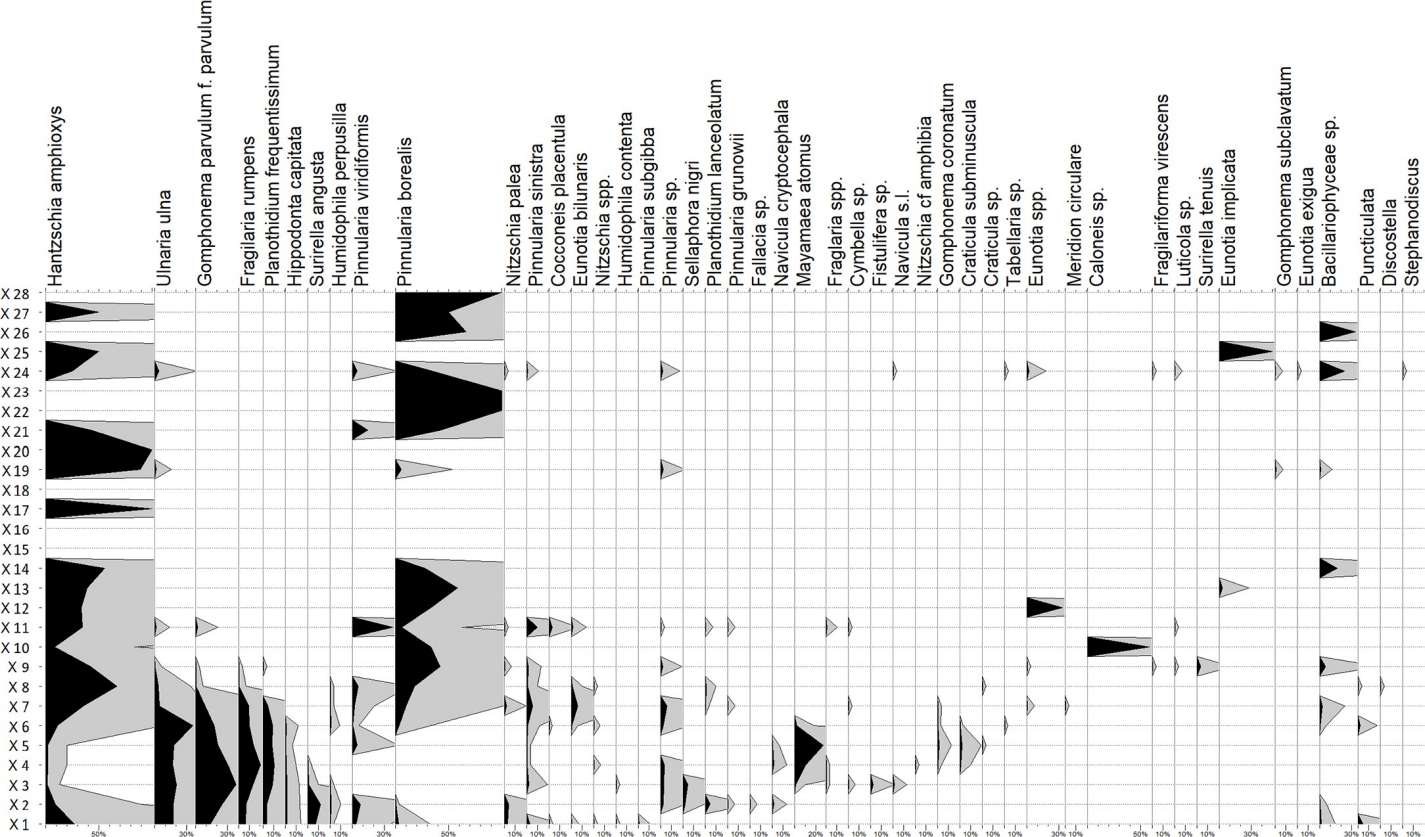
Bibracte/Mont Beuvray—La Chaume (ditch UF180). Percentage diatom diagram.

**Fig 14:**
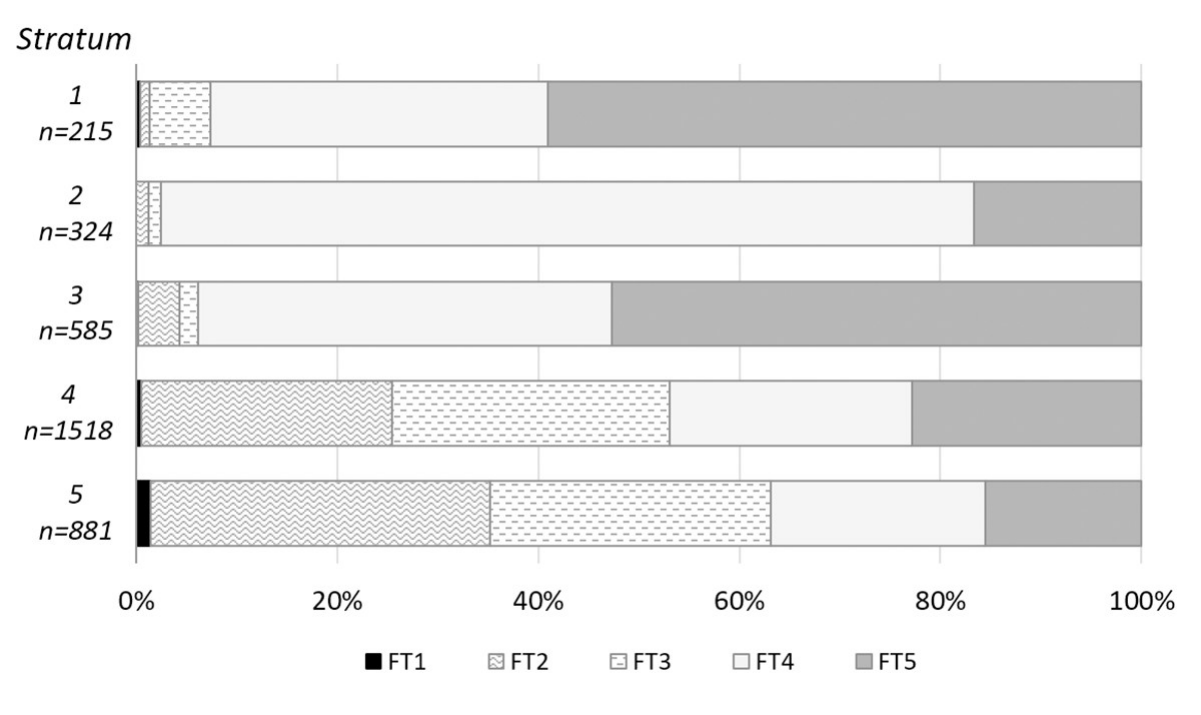
Bibracte/Mont Beuvray—La Chaume (ditch UF180). Functional trait representation in individual strata.

The water diatoms present in strata 5 and 4 (samples X1-X7) include representatives of all five FT groups (Fig 13)–especially FT2 and FT3 taxa were abundant (Fig 14). The periphytic diatoms (like the most abundant *Gomphonema parvulum* and *Planothidium frequentissimum*) live attached to the substrate with mucilaginous stalks, pads or films. *Ulnaria ulna* and *Fragilaria rumpens*, the most abundant representatives of the FT3 group, are araphid pennate diatoms with an alternating lifestyle (plankton/benthos). Categories FT4 and FT5 were present in all five strata; but the species richness of these two groups was much higher in strata 4 and 5. The abundance of FT4 and FT5 in strata 1–3 was mainly made up of aerotolerant terrestrial diatom species *Hantzschia amphioxys* and *Pinnularia borealis*.

### Parasitology

Parasite eggs were detected in 16 of the 33 samples (48.5%, Table 8). The commonly detected parasites were nematode helminths *Ascaris* and *Trichuris*. Both are faecal-oral parasites infecting humans. These helminths have frequently been reported in a variety of archaeological sites, e.g., [28, 67]. The detected quantities were relatively low and indicate that the ditch was not used as a primary deposit for faecal material or animal dung. The cestode (tapeworm) *Diphyllobothrium*, which is transmitted by the consumption of raw or undercooked freshwater fish, was present in two samples.

**Table 8:** Bibracte, La Chaume, ditch UF180. Extrapolated estimates of parasite densities by genus.

Stratum	Sample ID	*Ascaris*	*Trichuris*	*Diphyllobothrium*
		[eggs/g]	[eggs/g]	[eggs/g]
1	X28	64.6	0.0	0.0
1	X27	125.0	0.0	0.0
1	X26	240.0	0.0	0.0
1	X24	0.0	111.1	0.0
1	X23	125.0	62.5	0.0
2	X19	96.4	0.0	0.0
2	X17	0.0	222.2	0.0
2	X16	181.8	0.0	0.0
2	X13	0.0	115.9	0.0
3	X12	0.0	62.5	0.0
4	X08	113.2	0.0	0.0
4	X07	59.7	59.7	0.0
4	X05	0.0	313.7	78.4
5	X01	52.0	52.0	0.0
4–5	G03	179.1	0.0	0.0
4–5	G02	0.0	115.9	58.0

## Discussion

The purpose or importance of seemingly empty spaces within fortified sites is a widely debated question [13, 15, 17]. The ditch in Bibracte enclosed such a space during the 1^st^ century BC, before it was filled in. The ditch that was identified in the geophysical surveys seemingly ran through an ‘empty space’ within the inner ramparts of La Terrase and Le Porrey at Bibracte. To investigate the intended use of the space, we applied a wide array of methods to elucidate the ‘life and death’ of the ditch. This multidisciplinary approach is novel and could set a baseline for future investigations of such seemingly empty spaces. It also raises further questions on central issues like water and waste management inside Iron Age fortified sites.

### Formation and filling of the ditch

The different strata reflect the process through which the ditch was filled in and provide valuable information about its surroundings. Key for the full understanding of this process proved to be the combination of the various proxies complementing and confirming each other, and therefore, providing a complex and robust basis for our understanding.

**Stratum 5** (Fig 3; Table 2) represents the primary fill of the ditch that formed during or shortly after its first use. The available evidence is not sufficient to determine the date at which the ditch was constructed. The lifetime of the ditch in its original function, i.e., before its main infilling, could have been very short [22] or fairly long when kept clean. This has been shown for Medieval ditches that existed for hundreds of years, evidenced both by archaeology and written sources [54, 68]. No traces of repairs or re-cutting have been identified in the La Chaume ditch. Its maintenance was probably managed only through burning off the vegetation, as suggested by the numerous microcharcoals of *Gramineae* present in the micromorphology and palynology. Neolithic rondel ditches, for example, did not contain any charcoal, and therefore, their maintenance must have been managed in a different way [22]. During the first stages of its use, the ditch of La Chaume, Bibracte, contained stagnant water as shown by the micromorphological and diatom analyses (Fig 10). The diatom species composition in strata 5 (and 4) indicates a body of standing water based on the several individuals of planktonic centric diatoms that were found there. We can certainly exclude rapidly flowing water due to the absence of typically rheophilous taxa. Moreover, larger species (such as abundant and frequent *Ulnaria ulna*) would not remain in rapidly running water. Stagnant (or slowly moving) water up to 60 cm from the bottom may have accumulated in the ditch during rains and the impermeable subsoil was able to hold the water for a relatively long period of time. The diatom species richness indicates a stable and well-developed water ecosystem. Both diatom and parasite analyses provide further evidence of relatively clean water without strong anthropogenic impact. The number of faecal-orally transmitted parasites was relatively low when compared with latrines or waste deposits where human faecal material was deposited (see in [28]). It is highly unlikely that the ditch was used as a sanitation sewer. The appearance of fill containing large amphorae and pottery fragments marks the moment at which the ditch had lost its original function. The pottery and amphorae date this change to 50–30 BC (TPQ), which is in agreement with the radiocarbon dates.

**Stratum 4** was formed intentionally by redeposited rubble, likely used to level the infill of the ditch, which was filled with stagnant water in the lower part. Aerotolerant diatoms begin to appear in the middle part of stratum 4 and dominate all samples up to the top. The sedimentation likely became more intense after the slag deposition. Above it, the infill already shows signs of colluviated material. The presence of the dirty clay coating and infillings along the aggregate granules in micromorphological samples (Fig 10) reflect the deposition of disturbed material. This indicates that the stage at which this material was deposited proceeded quite rapidly. The lower strata (4 and 5) of the ditch fill with less eroded, large size pottery fragments were formed around the time of its use or shortly afterwards because the sherds are too large to be a part of a ‘cultural layer’ and there are too few matching fragments to suggest primary waste.

**Stratum 3** reflects a period of stable environment when the ditch was levelled and its surface was overgrown by vegetation. Pedogenesis had started and a humic A-horizon developed. This development may have resulted in the low influx of charred plant matter. Diatom abundance and species richness was low due to decreasing humidity [69]; however, a surprisingly high diatom abundance and diversity occurred in samples X11, X19 and X24 where water diatoms appeared again, possibly caused by short-term flooding such as a pool of rainwater. With time, the infill of the ditch was compressed due to subsidence and a linear depression indicating the original course of the ditch probably appeared. This depression was filled with the material making up stratum 2.

**Stratum 2** revealed the presence of building material, which indicates that the ditch served only as a dump area during this period. The building activities may have been connected to construction in the sanctuary premises in the vicinity, either during the Augustan period or, at the latest, during the 1^st^ century AD [70]. The absence of diatom valves in samples X15, X16 and X18 may have been caused by a completely desiccated environment that was probably connected to the presence of building waste. It is unlikely that the ditch was being filled with the intention of levelling the ground as we can distinguish thin dump layers as well as soil horizons with clear signs of pedogenetic processes.

**Stratum 1** formed on top of an already filled-in ditch and contains traces of activities of the Gallo-Roman (1^st^-3^rd^ century AD), Medieval and Post-Medieval periods. Written sources document annual markets during the High Medieval and Post-Medieval periods (from 1236 AD according to written sources [33]), but markets may have also taken place there much earlier. The reappearance of people and their lower hygiene standards are evident in an increase in parasite amounts and the spectrum of crops (millet and rye), which is different from the Iron Age taxa and more typical of the Medieval period in the region (Table 5; [63]). The number and/or size of fields in the vicinity increased, as ascertained by palynology (Fig 12). During these periods, traces of the ditch had already completely disappeared from the relief.

Regarding the radiocarbon dating, two issues have emerged. First, redeposited sediments (strata 2, 3 and 4) and the small size of the ^14^C samples make sample mobility a serious concern. This mobility prevents us from justifiably tying the measured ^14^C determinations with the formation or deposition times of the individual strata. Accordingly, the dated La Tène ^14^C samples *per se* only indicate agricultural activities. The second obstacle to a conclusive use of radiocarbon for archaeological ends is the atmospheric ^14^C production in the periods of habitation suggested by the pottery (120–50 BC and 50–30 BC; Table 2). Individual ^14^C dates for archaeological events from 120–30 BC will give large ranges of calibrated dates incapable of answering questions of subtle intra-site chronological development (IntCal13: [44]). The La Tène period cereal grains extracted from the ditch do not inform directly about the formation or deposition of ditch strata. These small and highly mobile ^14^C samples in redeposited sediments and atmospheric ^14^C production over the last two centuries BC have yielded results without sufficient chronological resolution. Nonetheless, the ^14^C dates from strata 2, 4 and 5 could be viewed as in line with the absolute chronology suggested by the artefacts present. The medians of the single phase model place the start of the habitation period at 159 cal BC and its end at 84 cal BC, although these dates are likely to be under- or overestimates of the true values. Conspicuously, not a single measured ^14^C determination has a value typical of 30 BC or later. The four cereal grains are from around 50 cal BC at the latest and only one may, but need not, be from 50 cal BC–4 cal AD (ETH-92301).

### Function of the ditch

Bibracte is a Late La Tène oppidum with strong Roman influence, which increased after 50 BC. Considering the probable date of the intentional filling of the ditch between 50–30 BC, the function of the ditch must be sought in both of these cultural milieus. V-shaped ditches are characteristic of the Roman fortification architecture in short term camps, documented frequently in Gaul [71] and beyond [72]. Roman military presence at oppida is evidenced by both written sources (for Bibracte cf. [31]) and archaeological records (e.g., Chaussée-Tirancourt: [73]; Titelberg: [74]). We still lack indisputable proof of Roman military presence at La Chaume, the top plateau of Bibracte oppidum in the vicinity of the ditch (cf. [75]), although according to written records Caesar spent the winter of 52/51 BC at Bibracte [31].

Ditches situated *intra muros* at Late Iron Age fortified sites (oppida) are usually either delimited or enclosed spaces with special functions or high importance for religious or public functions, or a combination thereof (e.g., Villeneuve-Saint-Germain: [76]; Titelberg: [13]), or served as water drains (Manching: [15]). The topography and geophysics of the studied area indicate that the ditch may be connected to the fortifications at La Terrasse and Le Porrey. It has previously been considered as two separate and individually fortified summits based on multiple palisaded ditches and a (partially) preserved talus that have been documented there for a long time [35, 36]. The geophysical survey made it clear that the La Chaume ditch joins the ditch surrounding La Terrasse (Fig 1), while its northern part is directed towards the fortification system of Le Porrey. The date of the enclosures at La Terrasse and Le Porrey is not well-established; the ditches there contain the 1^st^ century BC finds, although by themselves they can be more ancient (cf. the 3^rd^ century BC radiocarbon dates from the La Terrasse rampart: [77]).

The ditch enclosing La Chaume and La Terrasse most probably enclosed a special area, possibly with a socio-sacral function. It can be regarded as a religious area, if we consider the continuity of sacred places and the presence of a 1^st^ century AD sanctuary in its vicinity, and/or a gathering place. Sanctuaries with a curvilinear enclosure and almost no constructions inside are known in Gaul from the 3^rd^ century BC (Mirebeau-sur-Bèze, Ribemont-sur-Ancre: [78]), and have also been described in oppida (Alésia: [79]; Titelberg: [13]). Although the results from the La Chaume ditch are limited regarding clarification of the function of the area it enclosed, the study has proved that the area was maintained as grassland (based on a majority of grassland pollen taxa), possibly with a deliberately planted grove of lime trees (based on the dominance of lime tree pollen, which naturally does not occur in the area), was not used for the cultivation of crops (absence of cereal pollen) and was kept clean with limited human and animal access (based on diatom and parasite analyses).

### The environment of the oppidum

In spite of intense research in Bibracte, we know very little about the environment *intra muros* [80]. The palynological analysis conducted during our excavation gives us some insight into the vegetation growing in the surroundings of the summit and allows us to reflect on the role of vegetation in oppidum urbanism. Diatom and parasite analyses were used in the oppidum for the first time and provided valuable information about hygiene levels and waste management.

The area in the vicinity of the ditch at the summit of Mont Beuvray seems to have been deforested during the entire period over which the ditch was being filled in. The presence of grass was clearly documented in both microcharcoals and pollen. The vegetation of the Bibracte oppidum as observed at the bottom of the ditch infill can be reconstructed as a secondary disturbed woodland without naturally dominant forest species such as *Fagus* or *Quercus*, which were generally the most abundant trees in the Morvan during the Late Holocene [65]. The most commonly represented woody plants (*Corylus* and *Tilia*) were probably present in the form of shrubs as the result of specific woodland management, solitary trees or a copse (higher pollen production would depend on the glade effect in open landscape: [81]). Interestingly, the common presence of *Tilia* [80] must have been due to deliberate planting or deliberate exclusion from deforestation inside the ramparts because its presence in regional pollen records is rare. The environment i*ntra* and *extra muros* (during the time when the ditch was in its primary use) was rich in *Corylus*, which was abundant both in the pollen and charcoals.

The charcoal assemblage recorded from the bottom part of the ditch infill shows the dominance of beech and mostly originates from discarded hearth cinders with a mixture of taxa collected nearby, based on the least effort mechanism for collection of household fuel. Part of the charcoal material may have originated from burned construction(s), but neither the assemblages from strata nor those from individual samples can be explained as only representing remnants of burned construction (e.g., wooden palisade). Ecologically the wood taxa reflect various types of forest. Apart from mixed beech-oak with hazel (and probably *Ulmus* and *Carpinus*), we see evidence of moisture-loving trees growing on the banks of streams or in wetlands (*Alnus*, *Salix*, *Populus*), trees grown in the unstable environment of ravines (*Acer*), ruderalised wet forests (*Sambucus*), and open (*Cytisus scoparius*) and slowly re-forested stands (*Betula*).

The pollen record in the upper part of the infill indicates medieval and post-medieval landscape structures when the vegetation was divided into forest, pasture and fields. The higher crop representation in pollen (compared with Iron Age strata) corresponds with the existence of local arable farming; despite poor soil quality, fields were present at Mont Beuvray until World War II.

The cleanliness of the water in the ditch at the time of its primary use, confirmed by both parasite and diatom analyses, shows that the area was kept clean and free of faecal accumulation. The issue of waste management (including biological waste) in the densely populated area (several thousand inhabitants are believed to have lived in Bibracte during 50–30 BC: [29]) has not yet been discussed for oppida.

The radiocarbon dates suggest that waste management changed sometime during the 1^st^ century BC. The sediments from strata 2 and 4 that filled-in the ditch contain pottery from the period before and after 50 BC but the plant macroremains (as part of household waste) are present only from the period before 50 BC. If not caused by sampling strategy, the explanation may instead be that the manipulation with cereals from the early Augustan period or slightly earlier (i.e., from around 50–30 BC), which eventually produced charred grains, either changed in connection with Romanisation or did not take place where the ditch sediments formed, perhaps as a result of the spatial reorganisation of the site.

The presence of the freshwater fish-transmitted tapeworm *Diphyllobothrium latum* is sometimes related to the spread of Roman eating habits further into transalpine Europe [82]. Although *D*. *latum* had already occurred in this area before the Roman conquest in Neolithic finds, it was considerably less common during the Bronze and Iron Ages [83, 84]. The changes in the occurrence of *D*. *latum* may have been due to the change in the availability of freshwater fish resources [28] or a change in dietary culture, which is also shown in Bibracte during 50–30 BC by the strong influence of Mediterranean types of pottery [85].

## Conclusions

1. The ditch was identified in the geophysical survey. No visible terrain relics were preserved. The ditch was filled in at the end of the 1^st^ century BC.
2. When in use, the ditch was filled with water. The depth of water reached up to 60 cm above the bottom of the ditch. Diatom and parasite analyses indicate that the water was relatively clean, stagnant or slowly moving. Pollen analyses revealed that there was grassland vegetation cover in the vicinity of the ditch with solitary bushes or trees. The occurrence of herbaceous micro-charcoal suggests that the ditch slopes were maintained by burning the vegetation, while parasitological analysis showed that the nearby area was almost devoid of excrement. The ditch was very likely connected to the fortification (rampart and ditch) system at La Terrasse, and as such delimited an area the function of which during the life-time of the Iron Age oppidum remains unclear for now.
3. The ditch probably lost its primary function and was filled in between 50 and 30 BC. The material used for the fill was transported from another area, which had been settled during the period between the end of the 2^nd^ century BC and the first half of the 1^st^ century BC. The correlation of the artefactual (pottery and metal finds) and ecofactual (seeds and charcoal) evidence and the AMS ^14^C dates suggests that there was a change in the management of household waste during the 1^st^ century BC, demonstrated by the absence of crop finds later than 50 BC. The filled-in ditch was subsequently overgrown by herbaceous vegetation and used as a walking surface. After the subsidence of the sediments, the newly formed depression was filled in again before the end of the 1^st^ century BC. Part of the fill was building rubble, probably connected with the construction of the nearby sanctuary/fanum complex in the Augustan period.
4. During the Middle Ages an annual fair and a market were regularly organised during the first week of May at La Chaume including the location of the former ditch. The increased presence of people (and their lowered hygiene standards) is evident from the increase in the amount of parasites. The spectrum of crops differed from that in the Iron Age (with taxa typical of the Medieval period in the region) and the number and/or size of arable fields in the vicinity increased.
5. The specific role of trees in the urban area of the oppidum must be considered. High percentages of lime (*Tilia* sp.) were detected repeatedly in the pollen spectra from *intra muros* areas and outside its natural distribution, which may indicate deliberate planting of this tree at Mont Beuvray.
6. The fills of the ditch(es) studied using a multi-proxy approach represent an important source of information about the history of human activities at oppida, especially outside the built-up areas, or areas delimiting seemingly ‘empty spaces’. At Bibracte this approach contributed to a better understanding of the nature of the area. It is clear that by the first half of the 1^st^ century BC, at the latest, the area of the summit plateau delimited by the ditch and rampart system was maintained open and without building structures. It was then covered by grassland (*sensu stricto*, not arable fields) that was not used for grazing. It is also likely that there was deliberate planting of selected trees. These results support a socio-sacral function of the area.

## Supporting information

S1 Table Bibracte/Mont Beuvray—La Chaume (ditch UF180). Database of the volume and weight of the stones in the analysed units from the excavation in 2018 (cf. Table 1 for stone size classes and Fig 4 for the layers).
(XLS)

S2 Table Bibracte/Mont Beuvray—La Chaume (ditch UF180). Characteristics of the pottery and amphorae from the excavation in 2018. For pottery/amphorae codes see [29, 38, 39].
(XLSX)

S3 Table Bibracte/Mont Beuvray—La Chaume (ditch UF180). List of all diatom taxa found in samples X1-X28 (300 diatom valves were counted in each sample, if present).
(XLSX)
